# Metadata Made Easy: Develop and Use Domain‐Specific Metadata Schemes by following the dmdScheme approach

**DOI:** 10.1002/ece3.7764

**Published:** 2021-06-25

**Authors:** Rainer M. Krug, Owen L. Petchey

**Affiliations:** ^1^ Department of Evolutionary Biology and Environmental Studies University of Zürich Zurich Switzerland

## Abstract

1. Metadata plays an essential role in the long‐term preservation, reuse, and interoperability of data. Nevertheless, creating useful metadata can be sufficiently difficult and weakly enough incentivized that many datasets may be accompanied by little or no metadata. One key challenge is, therefore, how to make metadata creation easier and more valuable. We present a solution that involves creating domain‐specific metadata schemes that are as complex as necessary and as simple as possible. These goals are achieved by co‐development between a metadata expert and the researchers (i.e., the data creators). The final product is a bespoke metadata scheme into which researchers can enter information (and validate it) via the simplest of interfaces: a web browser application and a spreadsheet.
2. We provide the R package dmdScheme (dmdScheme: An R package for working with domain specific MetaData schemes (Version v0.9.22), 2019) for creating a template domain‐specific scheme. We describe how to create a domain‐specific scheme from this template, including the iterative co‐development process, and the simple methods for using the scheme, and simple methods for quality assessment, improvement, and validation.
3. The process of developing a metadata scheme following the outlined approach was successful, resulting in a metadata scheme which is used for the data generated in our research group. The validation quickly identifies forgotten metadata, as well as inconsistent metadata, therefore improving the quality of the metadata. Multiple output formats are available, including XML.
4. Making the provision of metadata easier while also ensuring high quality must be a priority for data curation initiatives. We show how both objectives are achieved by close collaboration between metadata experts and researchers to create domain‐specific schemes. A near‐future priority is to provide methods to interface domain‐specific schemes with general metadata schemes, such as the Ecological Metadata Language, to increase interoperability.

## Graphical abstract

The article describes a methodology to develop, enter, and validate domain specific metadata schemes which is suitable to be used by nonmetadata specialists. The approach uses an R package which forms the backend of the processing of the metadata, uses spreadsheets to enter the metadata, and provides a server based approach to distribute and use the developed metadata schemes.

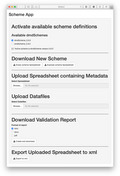

## 1 INTRODUCTION

To define a kind of gold standard for data handling, Wilkinson et al. (2016) developed the FAIR data principles. These define principles to make the data **F**indable, **A**ccessible, **I**nteroperable, and **R**eusable and help to assess data handling workflows in regard to openness.

There are multiple reasons why data should be widely reusable (Bishop & Kuula‐Luumi, 2017; Heaton, 2008; Pasquetto et al., 2017). Widely reusable means that anyone making reasonable efforts could reuse the data and that this would be the case even if the data creator(s) are unavailable. “Anyone” includes the creator(s) of the data, other members of the creating research group, and any other researcher. Use cases include using data from previous experiments to plan new ones, reanalyzing data using different or new preprocessing or analytical approaches to either compare different methodologies (Dufour & Richard, 2019) or to address new scientific aspects (e.g., the use of trait databases Schneider et al. (2019)), meta‐analyses (e.g., Culina et al., 2018; Zimmerman, 2008), reproduction of the studies, and use of data for teaching and training (e.g., Atenas and Havemann (2015); or Henty (2015)).

In order to being able to reuse data, it needs to be findable, it needs to be understandable why it was collected and how it was generated, it needs to be understandable which datasets are which, it needs to be understandable which variables contain what information, and relationships among variables must be specified (e.g., Gregory et al., 2019, 2020; Zimmerman, 2007, 2008). All this information should be stored in metadata; thus, metadata are essential for reuse (Gregory et al., 2020; Zimmerman, 2007). Furthermore, interoperability (the I of FAIR) requires standardized metadata schemes.

Metadata schemes have been developed which aim at providing a standardized structure and vocabulary to be used when providing the metadata. Examples of these schemes are the (meta)data standard Darwin Core (Darwin Core task group, 2014, for the current version please see http://rs.tdwg.org/dwc/) and the metadata standard Ecological Metadata Language (short EML) (Jones et al., 2019) in the field of biology/ecology, or more broadly Dublin Core (‘Dublin Core’, 2020). Interoperability is essential for research that relies on combining different datasets and is particularly important for data‐based interdisciplinary research as this very often combines data from different sources.

Given such important reasons for accompanying data with appropriate metadata, why do numerous datasets recently published not include useful metadata (Roche et al., 2015)? To have the metadata available requires the producer of the data to provide it. Therefore, the answer to the question of why many datasets are deposited without rich metadata is that the data creators have not prioritized creating rich metadata. There is some interest and some level of prioritization (e.g., Campbell et al. (2018) showed that especially early career researcher are participating in curating and sharing their data and metadata), but the uptake needs to be accelerated. A critical question that follows is how to motivate the creation and deposition of appropriate metadata. There are multiple possible answers; one that we focus upon is that creating metadata is not easy and creating metadata that conforms to a specific scheme is daunting and difficult for researchers. These schemes are relatively complex, as they are not specific to a research domain (see glossary for definition of “research domain”), but rather for a broad field. An example is the EML metadata scheme (Jones et al., 2019) which caters for earth and environmental science, while for domains in this field, not all properties of the EML scheme might be applicable. The advantages of being applicable to a broad field of science (e.g., consistent search across a range a wider range of domains, standardized property names, and vocabulary for metadata provision, interoperability) comes with the cost of being somewhat complex and rather difficult to understand, which could represent a significant barrier to use by research scientists not working in the field of metadata development.

Our aim was to make the processs of creating metadata not only easy, but also useful for the researcher that created the data and, if at all possible, a quite pleasurable experience to create. We follow the suggestion of Poisot et al. (2019), that **domain‐specific metadata schemes** (small and purpose‐built schemes) can be part of the solution to make ecological data easier to find and reuse. The example we use to illustrate a domain‐specific metadata scheme is from the research domain we term “Experimental Microbial Ecology” (e.g. Worsfold et al., 2009; Pennekamp et al., 2017; Altermatt et al., 2015) (hereafter EME). We chose this domain because of our familiarity with it and the fact that the data involved can be quite complex. Many measurements are often taken using different methods. Multiple treatments are often applied. Numerous taxa are often involved. Various steps of data processing are required to obtain analysis‐ready data (e.g., see Garnier et al., 2020; Pennekamp et al., 2017) from the measured raw data. The methods used can create large amounts of data (several terabytes). Therefore, EME is a sufficiently complex domain to be used as an illustration.

In this paper, we present as a case study the experience and results of our research group in developing the EME domain‐specific metadata scheme. We first used the R package dmdScheme (Krug & Petchey, 2019a) to create a template domain‐specific metadata scheme and then customized the template scheme to create the EME scheme (emeScheme (Krug & Petchey, 2019b). We end with a discussion on how these domain‐specific metadata schemes can be integrated into larger metadata schemes by using the example of EML (Jones et al., 2019).

The content of this article focuses on presenting the approach by which a domain‐specific metadata scheme can be created using the dmdScheme (Krug & Petchey, 2019a) R package, and its advantages in bringing domain‐specific metadata schemes to more domains and to facilitate the provision of rich and quality assured metadata. This article is supported by two Vignettes: one describes the dmdScheme and is aimed at developers of new domain‐specific schemes and at users interested in a more detailed description of the package. The other vignette is aimed at users of the emeScheme and could be modified for users of other domain‐specific schemes. Both are included in the Supplementary information of this article; updated versions are within the respective R packages.

## 2 RELEVANT FEATURES OF GOOD METADATA SCHEMES

**Standards**: A general consideration when developing domain‐specific metadata schemes, has to be to prevent the proliferation of a multitude of schemes, risking little or no interoperability among domains. To increase interoperability, each domain‐specific scheme should be as much as possible linked formally to standardized metadata schemes. A domain‐specific metadata scheme can be an easy‐to‐use interface to a more general and standardized metadata scheme. The approach described in this paper, the dmdScheme approach, contains infrastructure which can facilitate this. Further aspects are discussed in detail below. Three other features of domain‐specific metadata schemes can increase motivation of researchers to use them: **co‐development, ease of use**, and **data/metadata validation**.

**Co‐development** by metadata experts and researchers in respective domains ensures that the scheme can be shaped by providing input to identify essential properties to be included in the metadata, and to exclude nonessential metadata. The goal then is to create a domain‐specific metadata scheme that fits that domain. Co‐development not only results in a better product, but the resulting “ownership” of these schemes by researchers is likely to increase motivation to use them, to advertise them, to provide input for further development, and to include them in teaching and training.

**Easy metadata entry** is highly desirable. It should not be technically difficult, and presumably the easier the better. To accomplish these design goals, we made a metadata entry system that includes only a web browser‐based application and a spreadsheet. The simplicity of these interfaces should keep the additional workload for the researchers as small as possible. Moreover, these methods of metadata entry can be common across domains, meaning that it is not necessary to teach or learn a different tool for each domain. Previously developed applications for easy metadata entry include Morpho, a data management tool for earth, environmental, and ecological scientists (https://knb.ecoinformatics.org/tools/morpho). It is not maintained anymore and has not seen any activity for the last 5 years (https://github.com/NCEAS/morpho). Nevertheless, it is open source and could be developed further by all interested parties. Unfortunately, we did not manage run it, presumably due to incompatibilities with the java versions required that we were unable to resolve. Therefore, we were not able to compare its feature set with the here presented approach.

**Validation** of data and metadata can help researchers increase the quality of their data and metadata, for example, by checking that variables in datasets contain the information they should and that they correspond to the stated experimental treatment and observations. Most large metadata schemes provide mechanisms for validating the metadata (e.g., EML in the R package EML (Boettiger & Jones, 2019)). These validations assess mainly the syntactical correctness of the metadata, for example, if all required fields are provided and if numerical values are in the allowed range (if ranges are specified). More detailed (contextual and contentual) validation can be provided for more specific situations or for smaller domains of research, that is, for domain‐specific metadata schemes.

The aim of a domain‐specific metadata scheme would be to fulfill all of these four features. Nevertheless, in some cases it will not be possible to fulfill all without compromises which are not acceptable for the aim of developing specific schemes. This becomes apparent when considering a domain for which very specific use metadata is needed which cannot be linked to any larger metadata scheme. In this case, one should aim at linking the discovery metadata to a general scheme while keeping the use metadata unlinked. If both can be mapped, the domain‐specific metadata scheme would be a frontend to provide metadata following a larger metadata standard, using terminology the researchers are familiar with.

## 3 The Template dmdScheme PACKAGE

The R package dmdScheme (Krug & Petchey, 2019a) forms the core of developing and using domain‐specific metadata schemes following the dmdScheme approach. It is normally hidden for the researcher/user of the domain‐specific metadata schemes and mainly of concern for the actual developer or power user of new metadata schemes.

The package contains all the base functionality needed to develop a new domain‐specific metadata scheme. It includes functionality to create a spreadsheet for entering the domain‐specific metadata, functionality to read the metadata from that spreadsheet, basic validation functions, and export functions to xml and templates needed to implement the export to EML. It is important to note, that the dmdScheme package itself should not be used to enter actual metadata. It only contains a template for a metadata scheme.

How to develop a new scheme and how to use the package is explained in detail in the accompanying vignette Develop and Use the dmdScheme which is included in the supplemental material of this article.

A second part of the dmdScheme approach is a repository of domain‐specific schemes (Krug, 2020). Here, any developed domain‐specific schemes can be deposited. The R package dmdScheme contains functionality to load the selected scheme from this repository and installs the accompanying R package in a temporary library. This arrangement makes it possible to use the scheme not only together with the R package dmdScheme, but also in other programming languages, if so desired.

## 4 CREATING A DOMAIN‐SPECIFIC METADATA SCHEME

### 4.1 Creating the emeScheme

The scheme emeScheme (Krug & Petchey, 2019b) was developed based on the dmdScheme (Krug & Petchey, 2019a) and is tailored for data from Experimental Microbial Ecology. The motivation to develop this metadata scheme was born out of the realization that for long‐term storage and retrieval following the FAIR data principles, metadata and data format standards are needed to be able to find and retrieve the data at any later stage and to be able to reuse it, even in the own research environment. Therefore, it was decided to develop a rich metadata scheme which would provide enough metadata to be able to find the data and to reuse it.

As discussed in the Introduction section, interoperability across domains requires common cross‐domain metadata schemes. The dmdScheme package already contains the basic structures to provide an export to EML xml format. But one of the basic requirements of doing so is linking of the domain‐specific metadata properties to, in the case of the emeScheme, the EML properties. Hence, considerations in the drafting of the emeScheme (Krug & Petchey, 2019b) and some additional constraints (i.e., only one measurement and extraction method per data file), make it possible to translate the emeScheme metadata into EML (The export into EML is planned for the next major release of the emeScheme package.).

If in the development of a new dmdScheme the larger metadata scheme is kept in mind, it is possible to use all the functionalities of the package dmdScheme as a frontend for providing metadata which is compliant with larger, more complex, metadata schemes. In the same way, other large metadata schemes could be used as the framework for the domain‐specific metadata schemes. This would bridge the gap between simple to understand domain‐specific metadata schemes on the one side and complex and difficult to understand but applicable to a large range of different domains metadata schemes.

An open exchange between the researchers and a programmer developing the scheme was essential in turning the emeScheme into a domain‐specific metadata scheme which will be used by researchers to create their metadata. Researchers were involved in the process of developing the emeScheme from the beginning. This included regular meetings to identify properties in the scheme which are missing, redundant, or not needed. Finally, the researchers were the first testers of the metadata scheme.

The process of developing the emeScheme involves the following steps:

1. Definition of objectives by researchers and developer. This included the objective of FAIR compliance, but also ease of use and validation functionality.
2. Creation of a first version of the scheme by the developer. Entering, by researchers, data from a set of diverse experiments within the domain. The diversity of experiments is important, as different experiments may require different metadata properties and even structures.
3. Discussion among researchers and developer experiences entering the metadata, highlighting missing, redundant, or not needed properties in the scheme, etc.
4. Revision of the scheme by the developer and return to step 2.
5. Finalizing and publishing the scheme definition package.

Based on initial discussions, the scheme included information about the experiment itself as well as about the different data sets resulting from different measurements and analysis methods as well as treatments during the experiment. This information about the experiment is valuable contextual metadata. To simplify the provision of the metadata and to avoid duplication of the experimental metadata, all metadata would be entered into one spreadsheet file (with multiple sheets), with any required assignment of metadata to individual datasets done automatically in the final stage of the metadata export.

**FIGURE 1:**
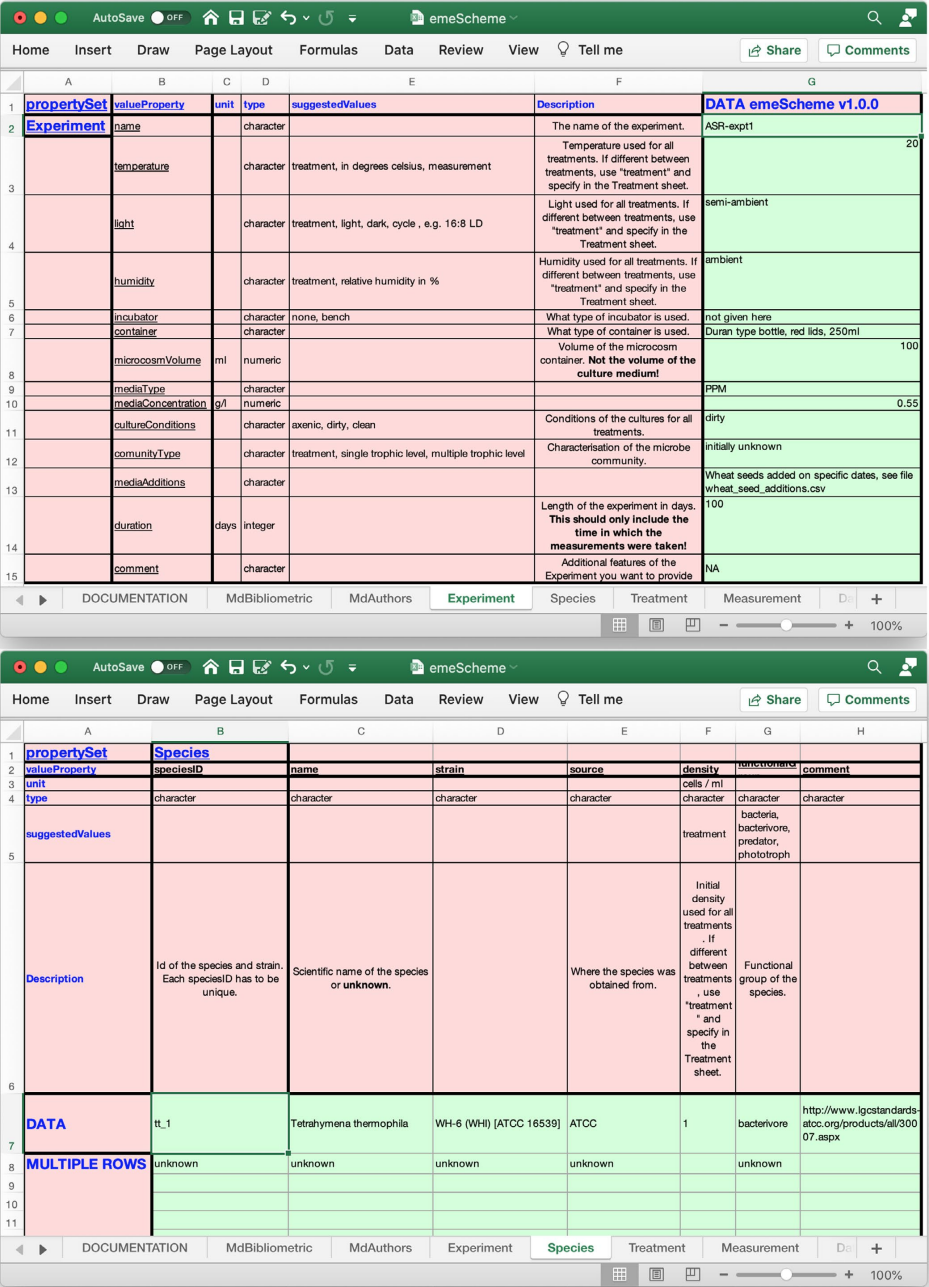
Two example sheets (Experiment and Species) in the emeScheme metadata file of the ‘emeScheme’ spreadsheet. The complete spreadsheet can be found in the supplemental material ‘emeScheme.xlsx’

This iterative process resulted in the spreadsheet emeScheme.xlsx (Figure 1 and Supplemental Material).

This scheme was then bundled together with additional examples and uploaded to the dmdScheme repository as emeScheme version 0.9.9 (Krug & Petchey, 2019b).

### 4.2 Enhancing the validation

Even though the package dmdScheme already contains a validation function, the validation is generic and mainly structural. The same applies for the export to xml, which only exports to a single xml file. Additional functionality in the emeScheme, that is, the contextual and contentual validation and the export of the metadata into one xml file per data file, is included in an accompanying emeScheme R package (Krug & Petchey, 2019b).

**FIGURE 2:**
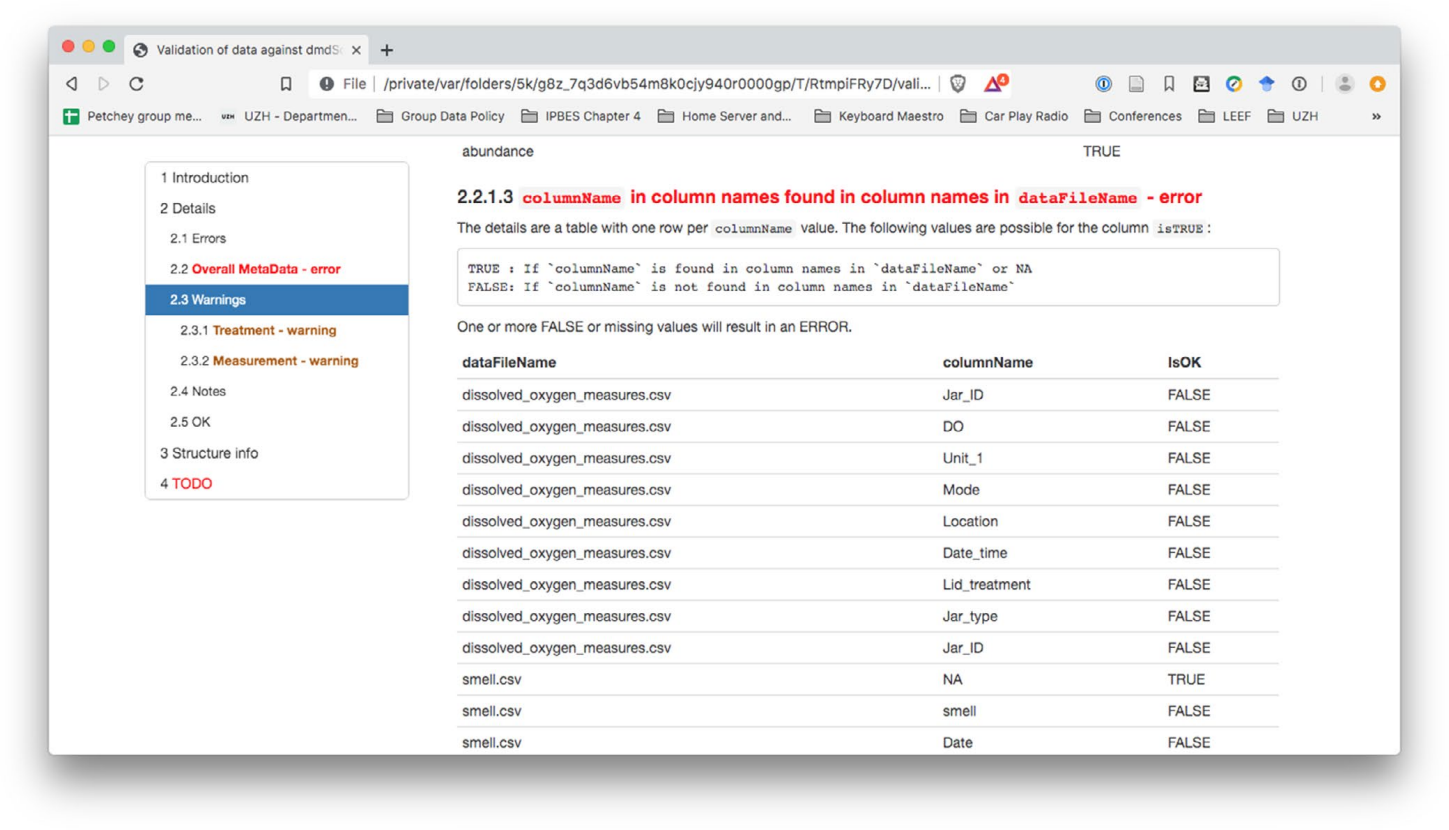
An example of the validation report. The full validation report is in the supplemental material file ‘Validation_Scheme.pdf’

Validation means the checking of the internal consistency of the metadata, compliance with the allowed and suggested values and types of the metadata as well as against the structure of the actual data files. This validation produces an html (see Figure 2), docx, or pdf report, which shows errors, warnings, or notes. Errors, warnings, and notes represent different levels of severity of detected faults or inconsistencies in the metadata. For example, if a value is not in the list of allowed values, it will result in an **error**, while if it is not in the list of suggested values, a **note** will be produced. The validation in the emeScheme package includes aspects not incorporated in the mainly structural and syntactical validation in the dmdScheme package. Therefore, it was necessary to write a new validation function to add the new validation rules, that is, the validation of the **structural metadata** which concerns the data files and its columns.

When the validation has completed without errors, the metadata can be exported to one xml file per data file. As in the package dmdScheme, the export to xml creates a single xml file, and we needed one xml file per data file, a new export function was included in the accompanying R package.

### 4.3 Using the emeScheme

**FIGURE 3:**
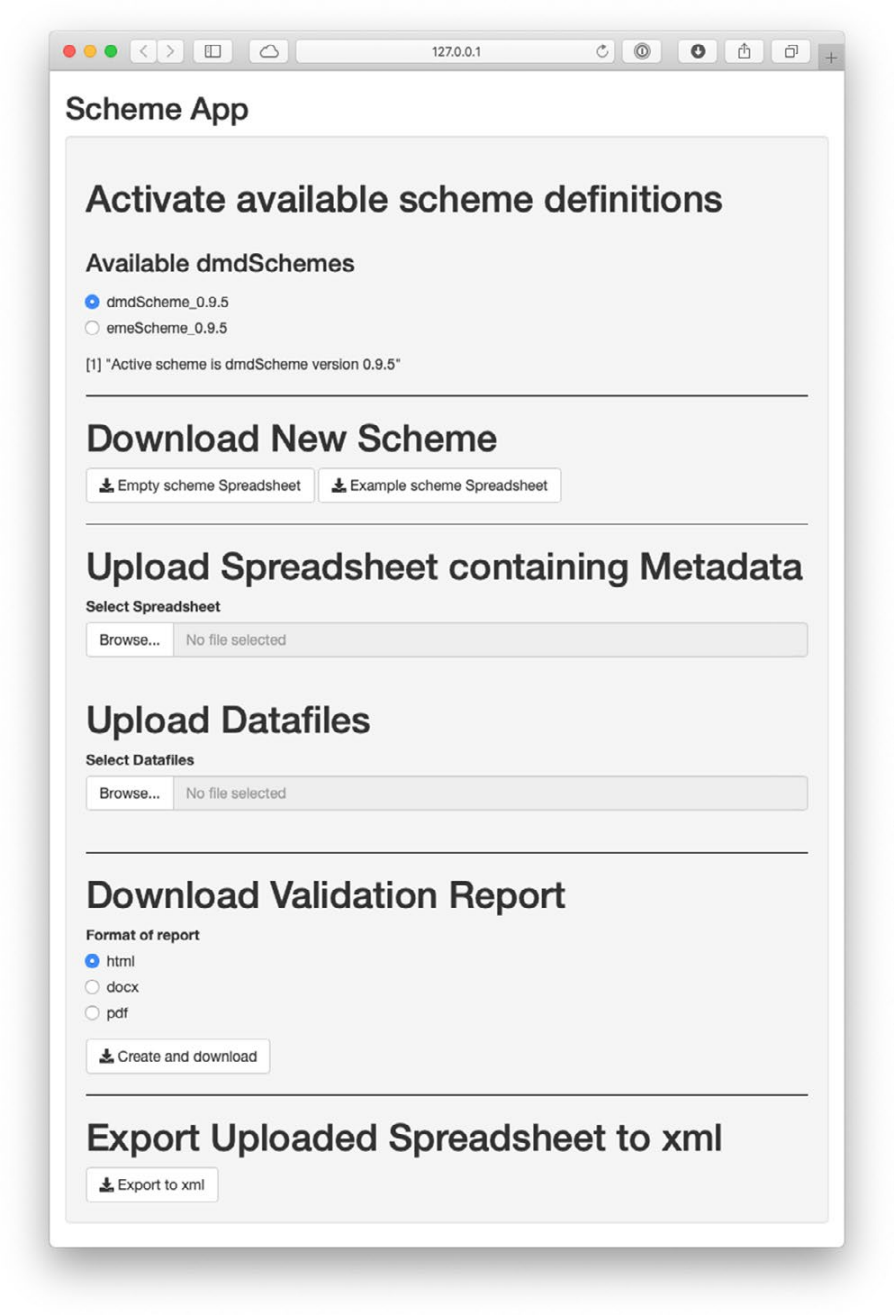
Web app to use the functionality of ‘dmdScheme’ derived metadata schemes. This app can be run as a https://rmkrug.shinyapps.io/dmd_app/ or also locally

The functionality in the emeScheme, actually of albftool dmdScheme derived metadata schemes, can be accessed by any of three approaches. As the scheme (and the accompanying R package) can be uploaded to the scheme repository (Krug, 2020), they are usable from a universal web app (Krug, 2019) (Figure 3). Each time the web app is started, it reloads a list of available scheme packages (and their accompanying R packages), and these can then be used in the app.

Even though this approach is the easiest, it requires the uploading of the metadata as well as the data to the server for validation. This might not be feasible because of confidentiality/privacy reasons or because of the large size of the data files. In this case, the app can also be launched from a local R session. The app then runs on the local computer and data never leaves the local computer.

As a third option, the emeScheme and all dmdScheme derived packages can also be used from the R command line.

## Glossary

- **Analysis**—processing the **analysis‐ready data** in order to address the research question.
- **Analysis‐ready data**—data ready for analysis; may be “ready” for a limited set of analyses. An example would be abundance of each of the species in a set of communities (e.g., population dynamic data of ecological communities). (Contrast with **raw data**.)
- **Data deposit package**—a collection of data and **metadata** files deposited in a long‐term repository. This consists of at least one data file and the **rich metadata** describing the data file(s) and associated information. May often contain multiple data files, each with its own **metadata** file.
- **Discovery metadata**—**metadata** which is useful for finding/discovering the data. This includes for example bibliometric **metadata**. It can also contain information about the species and location. In specialized repositories, this **metadata** can be more complex and contain more properties (e.g. GBIF (2020) which uses the EML metadata scheme (Jones et al., 2019)), than for example in Zenodo (2020) which is using a general metadata scheme (DataCite, 2021). **Discovery metadata** should be indexed and be available to a search engine. The scheme describing this **metadata** is usually given by the repository.
- **Domain/research domain**—a grouping of e.g. experiments, research, and/or questions addressed, whose data sets can be described using **metadata** following one **metadata scheme** which can be regarded as **rich metadata**. One example is “Experimental Microbial Ecology” for which the metadata scheme *emeScheme* (Krug & Petchey, 2019b) was developed. Fields, such as Ecology and Evolutionary Biology, contain numerous domains.
- **Domain‐specific metadata scheme**—a **metadata scheme** for a **domain**.
- **FAIR data principles**—guiding principles to make data Findable, Accessible, Interoperable, and Reusable (Wilkinson et al., 2016).
- **Field‐specific metadata scheme**—a **metadata scheme** general and broad enough to apply to an entire field. For example the Ecological Metadata Scheme (EML) (Jones et al., 2019).
- **Long‐term storage/preservation**—the process of having data stored/preserved and accessible for the long‐term (i.e., greater than 20 years envisaged).
- **Long‐term (storage)** **repositories**—repositories which offer **long‐term storage**. Examples are Zenodo (2020) or DRYAD (2021). The Zenodo repository currently has plans defined for at least 20 years of operation.
- **Metadata**—data about data. Metadata can be as little as the name of a variable/column in a spreadsheet of data, though such limited **metadata** would likely not be considered **rich metadata** and may not make the data **FAIR**. **Metadata** can be assigned two nonexclusive aspects, namely **discovery metadata** and **use metadata**.
- **Metadata scheme**—a formalized description of the **metadata** to be included in, for example, a **data deposit package**, their formats, and which ones are compulsory or not. A formal scheme assists with the indexing of the **metadata** that is required for programmatic searching and extracting **metadata** and data from repositories.
- **Preprocessing**—the preparation of the **raw data** to make it **analysis‐ready**. This should be done by a script to make the process reproducible and may use different parameters/methods which need to be adjusted based on the research question and the **raw data**.
- **Raw data**—data as provided by the measuring device. This could be images or videos taken from a camera, tables as returned from machines or hand‐written records.
- **Rich metadata**—defined by the Research Data Alliance (Research Data Alliance, 2017) as “data with enough accurate and relevant attributes to make it easily findable.”
- **Use metadata**—**metadata** which is useful/essential to be able to (re)use the data. In its most basic form, this is information containing the column names and description of the data files. It should also contain information about the experimental layout, approach, and data. This **metadata** can be described either by the **metadata scheme** used by the repository (GBIF (2020) uses the EML metadata scheme (Jones et al., 2019) which includes **use metadata**) or as an additional **metadata** file as defined by, for example, a **domain‐specific metadata scheme**. These data do not have to be indexed.

## CONFLICT OF INTEREST

The authors declare that they have no conflict of interest.

## AUTHOR CONTRIBUTIONS

**Rainer M. Krug:** Conceptualization (lead); methodology (equal); software (lead); writing‐original draft (lead). **Owen L. Petchey:** Conceptualization (supporting); methodology (equal); writing‐original draft (supporting).

### OPEN RESEARCH BADGES

This article has earned an Open Materials Badge for making publicly available the digitally‐shareable data necessary to reproduce the reported results. The data is available at https://doi.org/10.5281/zenodo.3894237 and https://doi.org/10.5281/zenodo.4529180s.

## Supporting information

Supplementary Material

Supplementary Material

Supplementary Material

Supplementary Material

## Data Availability

The package does not use any data. The code is available as followed: dmdScheme Package: The package is available on github at https://github.com/Exp‐Micro‐Ecol‐Hub/dmdScheme. The version used in this paper (v1.2) has the doi https://doi.org/10.5281/zenodo.3894237. emeScheme Package: The package is available on github at https://github.com/Exp‐Micro‐Ecol‐Hub/emeScheme. The version used in this paper (v1.1.7) has the doi https://doi.org/10.5281/zenodo.4529180.

## References

[ece37764-bib-0001] Altermatt, F. , Fronhofer, E. A. , Garnier, A. , Giometto, A. , Hammes, F. , Klecka, J. , Legrand, D. , Mächler, E. , Massie, T. M. , Pennekamp, F. , Plebani, M. , Pontarp, M. , Schtickzelle, N. , Thuillier, V. , & Petchey, O. L. (2015). Big answers from small worlds: A user's guide for protist microcosms as a model system in ecology and evolution. Methods in Ecology and Evolution, 6(2), 218–231. 10.1111/2041-210X.12312

[ece37764-bib-0002] Atenas, J. , & Havemann, L. (2015). Open data as open educational resources: Case studies of emerging practice. 10.6084/m9.figshare.1590031.v1

[ece37764-bib-0003] Bishop, L. , & Kuula‐Luumi, A. (2017). Revisiting qualitative data reuse: A decade on. SAGE Open, 7(1), 2158244016685136. 10.1177/2158244016685136

[ece37764-bib-0004] Boettiger, C. , & Jones, M. B. (2019). EML: Read and write ecological metadata language files.

[ece37764-bib-0005] Campbell, H. A. , Micheli‐Campbell, M. A. , & Udyawer, V. (2018). Early career researchers embrace data sharing. Trends in Ecology & Evolution, 34(2), 95–98. 10.1016/j.tree.2018.11.010 30573193

[ece37764-bib-0006] Culina, A. , Crowther, T. W. , Ramakers, J. J. C. , Gienapp, P. , & Visser, M. E. (2018). How to do meta‐analysis of open datasets. Nature Ecology & Evolution, 2(7), 1053–1056. 10.1038/s41559-018-0579-2 29915339

[ece37764-bib-0007] Darwin Core task group, B. I. S. (TDWG) (2014). Darwin Core: 2014‐11‐08. Zenodo. 10.5281/ZENODO.12694

[ece37764-bib-0008] DataCite Metadata Scheme (2021). https://schema.datacite.org

[ece37764-bib-0009] DRYAD (2021). https://datadryad.org/stash

[ece37764-bib-0010] Dublin Core: Metadata Terms (2020). https://www.dublincore.org/specifications/dublin‐core/dcmi‐terms/

[ece37764-bib-0011] Dufour, I. F. , & Richard, M.‐C. (2019). Theorizing from secondary qualitative data: A comparison of two data analysis methods. Cogent Education, 6(1), 1690265. 10.1080/2331186X.2019.1690265

[ece37764-bib-0012] Garnier, A. , Hulot, F. D. , & Petchey, O. L. (2020). Manipulating the strength of organism‐environment feedback increases nonlinearity and apparent hysteresis of ecosystem response to environmental change. Ecology and Evolution, 10(12), 5527–5543. 10.1002/ece3.6294 32607172 PMC7319241

[ece37764-bib-0013] GBIF (2020). https://www.gbif.org/

[ece37764-bib-0014] Gregory, K. , Groth, P. , Cousijn, H. , Scharnhorst, A. , & Wyatt, S. (2019). Searching data: A review of observational data retrieval practices in selected disciplines. Journal of the Association for Information Science and Technology, 70(5), 419–432. 10.1002/asi.24165 31763358 PMC6853156

[ece37764-bib-0015] Gregory, K. , Groth, P. , Scharnhorst, A. , & Wyatt, S. (2020). Lost or found? Discovering data needed for research. Harvard Data Science Review, 2(2). 10.1162/99608f92.e38165eb

[ece37764-bib-0016] Heaton, J. (2008). Secondary analysis of qualitative data: An overview. Historical Social Research/Historische Sozialforschung, 33(3(125)), 33–45.

[ece37764-bib-0017] Henty, M. (2015). Teaching with research data (report to the Australian National Data Service (ANDS)) (p. 37).

[ece37764-bib-0018] Jones, M. , O'Brien, M. , Mecum, B. , Boettiger, C. , Schildhauer, M. , Maier, M. , Chong, S. (2019). Ecological metadata language version 2.2.0. 10.5063/f11834t2

[ece37764-bib-0019] Krug, R. M. (2019). dmdScheme App. https://rmkrug.shinyapps.io/dmd_app/

[ece37764-bib-0020] Krug, R. M. (2020). dmdSchemeRepository. https://github.com/Exp‐Micro‐Ecol‐Hub/dmdSchemeRepository

[ece37764-bib-0021] Krug, R. M. , & Petchey, O. L. (2019a). dmdScheme: An r package for working with domain specific MetaData schemes (Version v0.9.22). 10.5281/zenodo.3581970

[ece37764-bib-0022] Krug, R. M. , & Petchey, O. L. (2019b). emeScheme: A package for working with ecological microbial experimental MetaData. Zenodo. 10.5281/zenodo.1544945

[ece37764-bib-0023] Pasquetto, I. , Randles, B. , & Borgman, C. (2017). On the reuse of scientific data. Data Science Journal, 16, 8. 10.5334/dsj-2017-008

[ece37764-bib-0024] Pennekamp, F. , Griffiths, J. I. , Fronhofer, E. A. , Garnier, A. , Seymour, M. , Altermatt, F. , & Petchey, O. L. (2017). Dynamic species classification of microorganisms across time, abiotic and biotic environments ‐ A sliding window approach. PLoS One, 12(5), e0176682. 10.1371/journal.pone.0176682 28472193 PMC5417602

[ece37764-bib-0025] Poisot, T. , Bruneau, A. , Gonzalez, A. , Gravel, D. , & Peres‐Neto, P. (2019). Ecological data should not be so hard to find and reuse. Trends in Ecology & Evolution, 34(6), 494–496. 10.1016/j.tree.2019.04.005 31056219

[ece37764-bib-0026] Research Data Alliance (2017). Rich Metadata ‐ DFT. https://smw‐rda.esc.rzg.mpg.de/index.php/Rich_Metadata

[ece37764-bib-0027] Roche, D. G. , Kruuk, L. E. B. , Lanfear, R. , & Binning, S. A. (2015). Public data archiving in ecology and evolution: How well are we doing? PLOS Biology, 13(11), e1002295. 10.1371/journal.pbio.1002295 26556502 PMC4640582

[ece37764-bib-0028] Schneider, F. D. , Fichtmueller, D. , Gossner, M. M. , Güntsch, A. , Jochum, M. , König‐Ries, B. , Le Provost, G. , Manning, P. , Ostrowski, A. , Penone, C. , & Simons, N. K. (2019). Towards an ecological trait‐data standard. Methods in Ecology and Evolution, 10(12), 2006–2019. 10.1111/2041-210X.13288

[ece37764-bib-0029] Wilkinson, M. D. , Dumontier, M. , Aalbersberg, I. J. J. , Appleton, G. , Axton, M. , Baak, A. , Blomberg, N. , Boiten, J.‐W. , da Silva Santos, L. B. , Bourne, P. E. , Bouwman, J. , Brookes, A. J. , Clark, T. , Crosas, M. , Dillo, I. , Dumon, O. , Edmunds, S. , Evelo, C. T. , Finkers, R. , … Mons, B. (2016). The FAIR Guiding Principles for scientific data management and stewardship. Scientific Data, 3, 160018. 10.1038/sdata.2016.18 26978244 PMC4792175

[ece37764-bib-0030] Worsfold, N. T. , Warren, P. H. , & Petchey, O. L. (2009). Context‐dependent effects of predator removal from experimental microcosm communities. Oikos, 118(9), 1319–1326. 10.1111/j.1600-0706.2009.17500.x

[ece37764-bib-0031] Zenodo ‐ Research. Shared (2020). https://zenodo.org/

[ece37764-bib-0032] Zimmerman, A. (2007). Not by metadata alone: The use of diverse forms of knowledge to locate data for reuse. International Journal on Digital Libraries, 7(1–2), 5–16. 10.1007/s00799-007-0015-8

[ece37764-bib-0033] Zimmerman, A. S. (2008). New knowledge from old data: The role of standards in the sharing and reuse of ecological data. Science, Technology, & Human Values, 33(5), 631–652. 10.1177/0162243907306704

