# Supplementary material for: Metadata Made Easy: Develop and Use Domain‐Specific Metadata Schemes by following the dmdScheme approach
Source: Ecol Evol. 2021 Jun 25;11(14):9174–81. doi: 10.1002/ece3.7764 (PMC8293710; doi:10.1002/ece3.7764)
Supplement: Supplementary file 1 — Supplementary Material [file ECE3-11-9174-s002.pdf]

Develop and use the `dmdScheme`

dmdScheme_0.9.9

Rainer M Krug Rainer.Krug@uzh.ch

2021-04-19

- Using the `dmdScheme`
  - Preparation
  - Select scheme
  - Download new scheme
  - Enter / Edit metadata on downloaded xlsx
  - Validate metadata and create report
  - Export uploaded spreadsheet to xml
- The R Package
  - Installation
  - Loading the package
  - Managing installed schemes
    - `scheme_default()`
    - `scheme_repo()`
    - `scheme_list_in_repo()`
    - `scheme_download()`
    - `scheme_install()`
    - `scheme_install_r_package()`
    - `scheme_list()`
    - `scheme_use()`
    - `scheme_active()`
    - `scheme_path_index_template()`
    - `scheme_path_xlsx()`
    - `scheme_path_xml()`
    - `scheme_uninstall()`
    - `scheme_installed()`
    - `scheme_make()`
- Entering new Metadata
  - Importing Data from Excel Sheet
  - Print dmdScheme Data
  - Validating your metadata
  - Converting Metadata to xml
  - Re-import from xml into R
- Developing new dmdSchemes derived scheme definitions
  - Create and edit new definition
  - Minimum requirements for metadata schemes derived from `dmdScheme`
- Developing accompanying R packages

Using the `dmdScheme`

The functionality for using the dmdScheme is available either via the online app (which can also be run on an in-house shiny server), the local running app, or via the R command line. The simplest method is using the online app, as no additional software needs to be installed locally. To be able to use the dmdScheme functionality locally, either via the app or via the R command line, it is necessary to install R [@RCoreTeam2019] and the `dmdScheme` package [@Krug2019a] in R.

In the following section, I will go through the different stages of using the `dmdScheme` via the web app, a local app or the R prompt. The detailed commands which have to be used can be found in Figure 1.

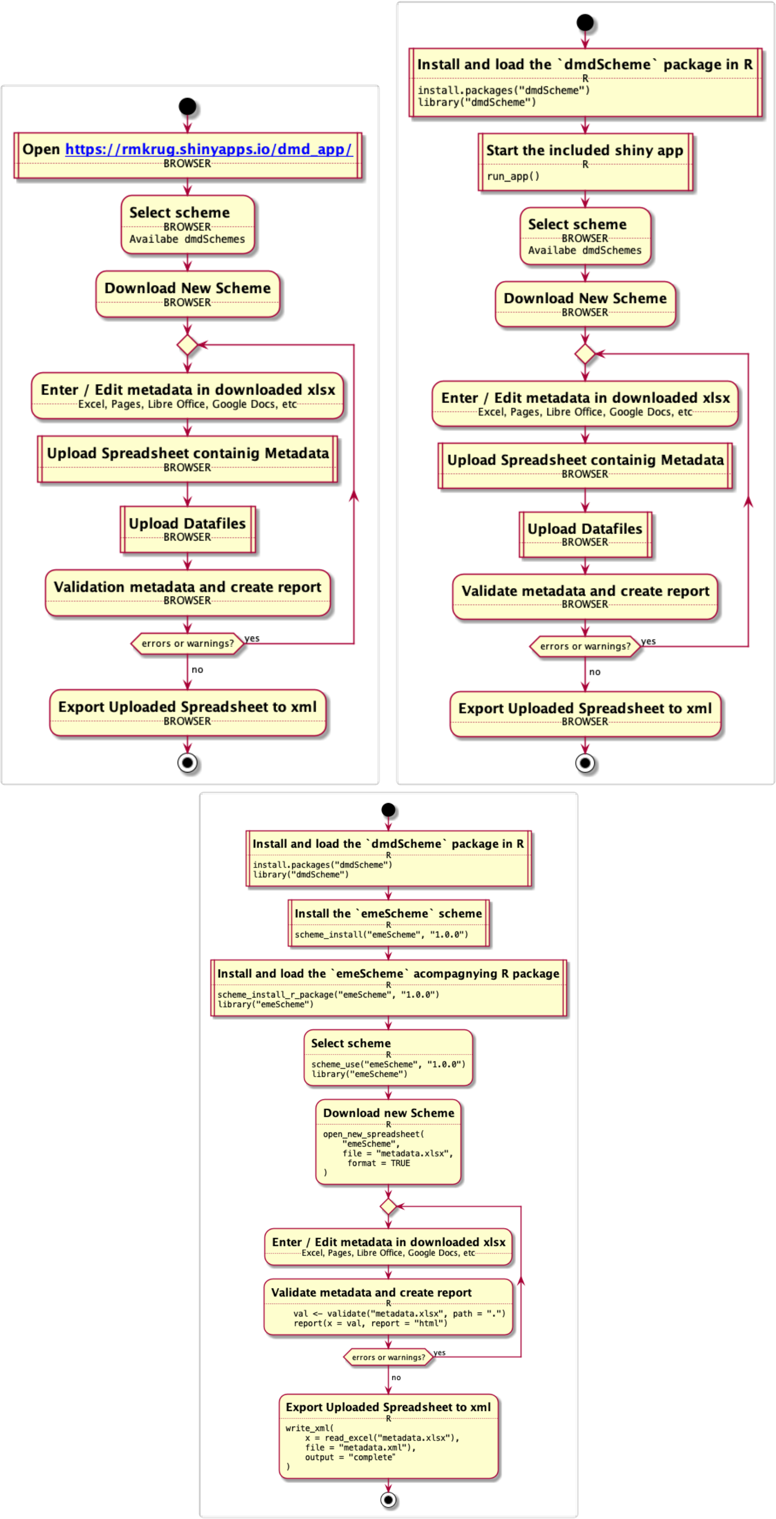

Figure 1: Workflow of using `dmdScheme` (A) via the web app, (B) via the app locally, (C) via the R prompt. Square boxes indicate steps which are not common to all three, rounded boxes indicate steps identical to the different ways, although how they are executed can differ.

Preparation

The app (Figure 2) can be accessed either via the internet as a web app at https://rmkrug.shinyapps.io/dmd_app/, or locally. To run it locally, you need R and the `dmdScheme` package installed. After loading the `dmdScheme` package, you can start the app locally by running `run_app()` at the R prompt. After these steps, the usage of the two apps is identical.

When using the `dmdScheme` from the command line, the initial setup is the same as running the app locally, only that it is not necessary to start the app.

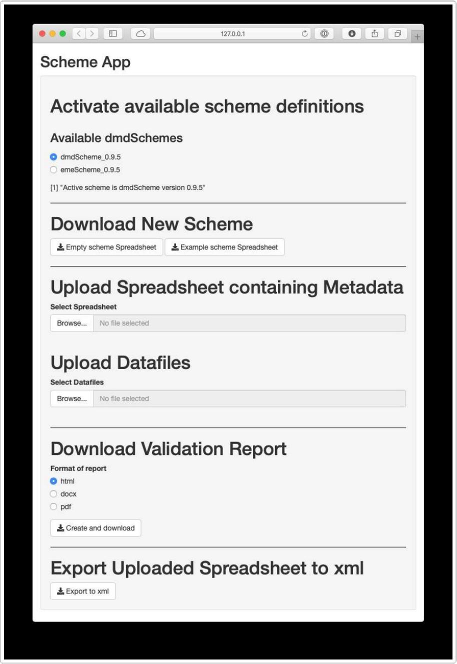

Figure 2: Screenshot of the dmdScheme app.

Select scheme

The package `dmdScheme` does not come with a specific scheme, and installs upon loading a generic dmdScheme from the dmdScheme scheme repository at https://github.com/Exp-Micro-Ecol-Hub/dmdSchemeRepository. In nearly all circumstances, a specific scheme needs to be installed, together with the accompanying R package. In the app, this is done via selecting a theme in the section "Available dmdSchemes". This list is populated automatically upon starting of the app from the schemes available in the scheme repository. The selection of the scheme will download the scheme definition package, install any accompanying R package as specified in the scheme definition package, load the accompanying R package, and activate the scheme definition.

To do this from the R prompt, one has do these steps manually and install the scheme, install the accompanying R package, load the accompanying R package, and activate the scheme itself (see Figure 1 C for the commands).

Download new scheme

The spreadsheet to enter the metadata can be obtained from the app via the "Empty scheme spreadsheet" button. This will download an `.xlsx` spreadsheet containing the definition of the scheme and the cells for the metadata which need to be filled in.

In R, the spreadsheet can be obtained by using the `open_new_spreadsheet()` command.

Figure 3 shows two screenshots of the spreadsheet as opened in Excel.

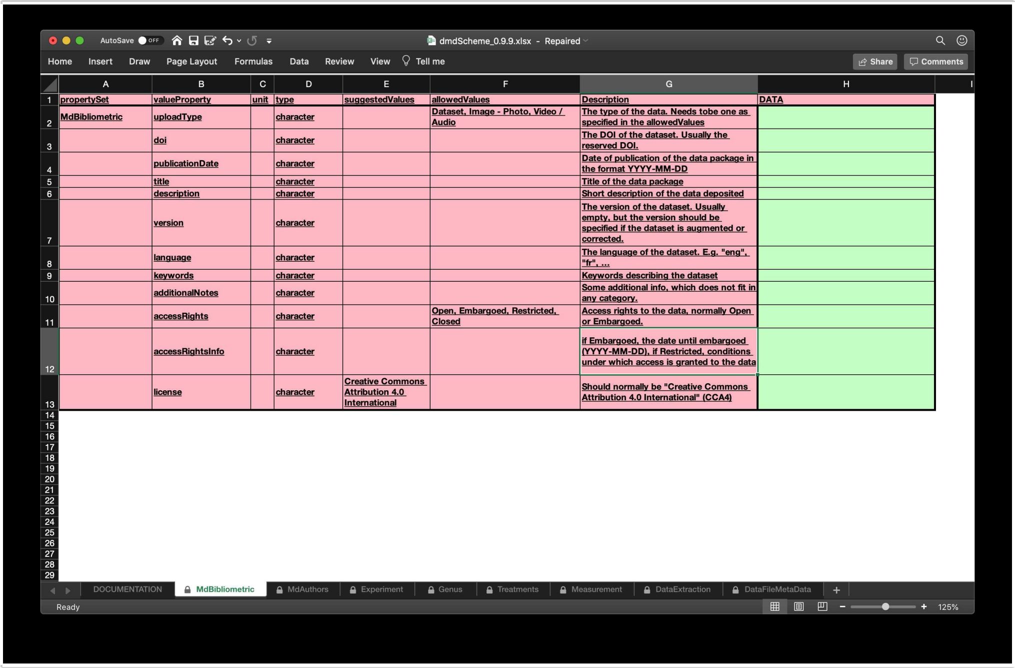

	A	B	C	D	E	F	G	H
1	propertySet	valueProperty	unit	type	suggestedValues	allowedValues	Description	DATA
2	MdBibliometric	uploadType		character		Dataset, Image - Photo, Video / Audio	The type of the data. Needs tobe one as specified in the allowedValues	
3		doi		character			The DOI of the dataset. Usually the reserved DOI.	
4		publicationDate		character			Date of publication of the data package in the format YYYY-MM-DD	
5		title		character			Title of the data package	
6		description		character			Short description of the data deposited	
7		version		character			The version of the dataset. Usually empty, but the version should be specified if the dataset is augmented or corrected.	
8		language		character			The language of the dataset. E.g. "eng", "fr", ...	
9		keywords		character			Keywords describing the dataset	
10		additionalNotes		character			Some additional info, which does not fit in any category.	
11		accessRights		character		Open, Embargoed, Restricted, Closed	Access rights to the data, normally Open or Embargoed.	
12		accessRightsInfo		character			if Embargoed, the date until embargoed (YYYY-MM-DD), if Restricted, conditions under which access is granted to the data	
13		license		character	Creative Commons Attribution 4.0 International		Should normally be "Creative Commons Attribution 4.0 International" (CCA4)	

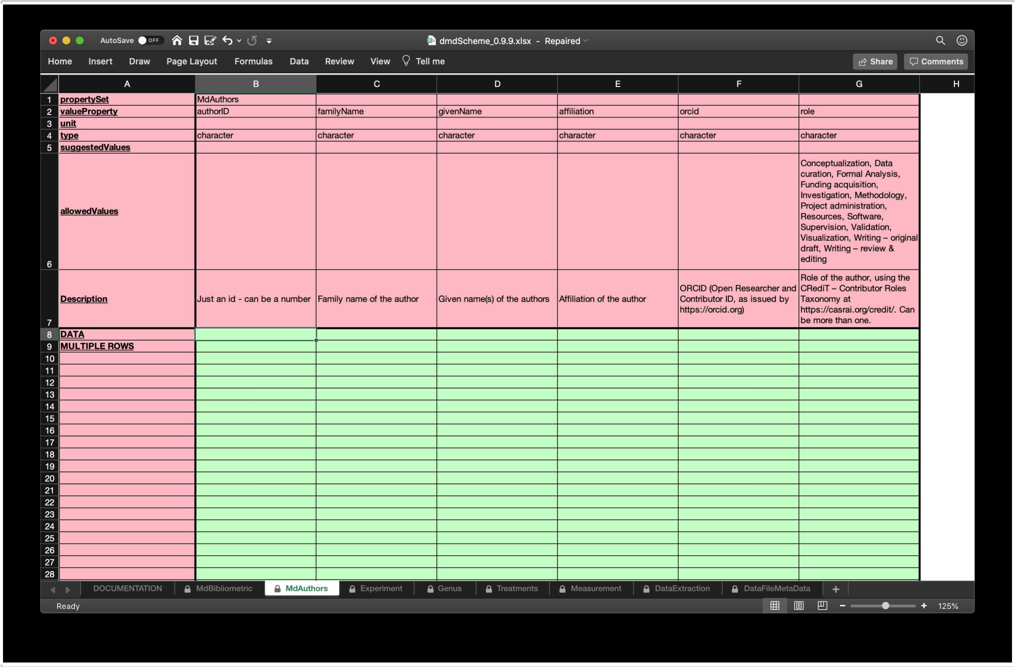

	A	B	C	D	E	F	G
1	propertySet	MdAuthors					
2	valueProperty	authorID	familyName	givenName	affiliation	orcid	role
3	unit						
4	type	character	character	character	character	character	character
5	suggestedValues						
6	allowedValues						Conceptualization, Data curation, Formal Analysis, Funding acquisition, Investigation, Methodology, Project administration, Resources, Software, Supervision, Validation, Visualization, Writing – original draft, Writing – review & editing
7	Description	Just an id - can be a number	Family name of the author	Given name(s) of the authors	Affiliation of the author	ORCID (Open Researcher and Contributor ID, as issued by https://orcid.org)	Role of the author, using the CRediT – Contributor Roles Taxonomy at https://casrai.org/credit/. Can be more than one.
8	DATA						
9	MULTIPLE ROWS						

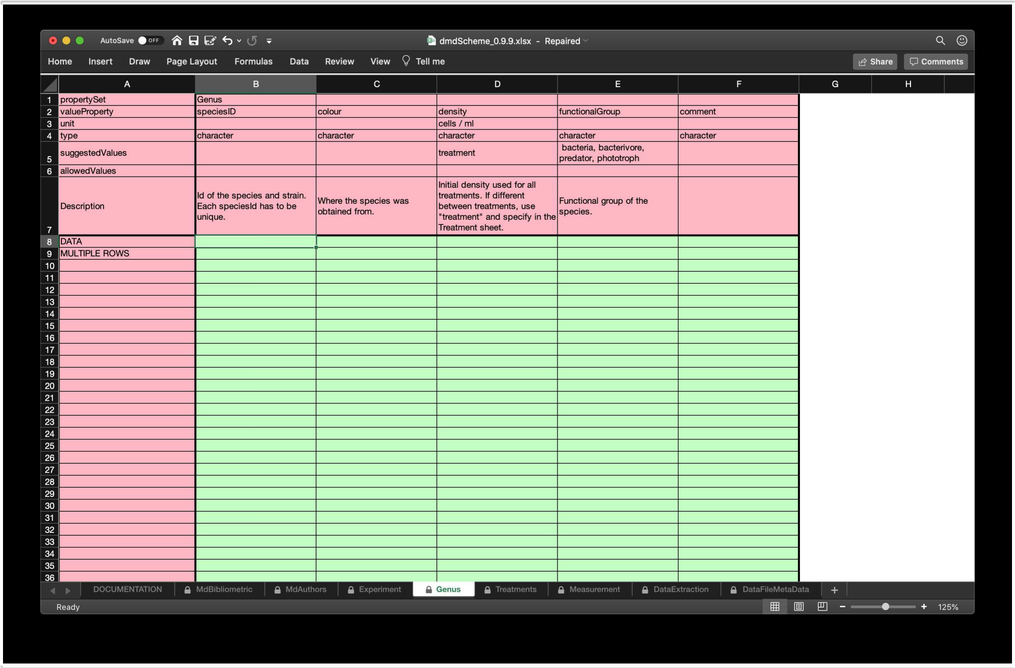

	A	B	C	D	E	F
1	propertySet	Genus				
2	valueProperty	speciesID	colour	density	functionalGroup	comment
3	unit			cells / ml		
4	type	character	character	character	character	character
5	suggestedValues			treatment	bacteria, bacterivore, predator, phototroph	
6	allowedValues					
7	Description	Id of the species and strain. Each speciesId has to be unique.	Where the species was obtained from.	Initial density used for all treatments. If different between treatments, use "treatment" and specify in the Treatment sheet.	Functional group of the species.	
8	DATA					
9	MULTIPLE ROWS					

Figure 3: Some example tabs from the `emeScheme` spreadsheet. The first to contains bibliometric metadata modelled along the requirements by DataCite and the authors in the second tab. The third one contains

metadata about the Species used in the experiment. The complete spreadsheet can be found in the supplemental material `emeScheme.xlsx`.

Enter / Edit metadata on downloaded xlsx

The entering of the metadata is done in the spreadsheet downloaded before. The spreadsheet contains the definition of the scheme as well as all information needed to fill in the metadata, including “type” of the metadata (e.g. numeric, character, or boolean), “allowed values” or “suggested values”, “unit” as well as a “Description” field.

Metadata can be entered in all green cells, while all red cells are (should be) locked and are part of the scheme definition.

Here the familiarity of researchers with spreadsheets plays an important role, as the technical details of the entering itself do not need to be explained as it is a standard spreadsheet.

The metadata in the `.xlsx` file can be entered and edited in likely all spreadsheet programs which can read and write `.xlsx` files. We tested successfully Excel, Pages, Libre Office and Google Docs.

A spreadsheet as frontend for entering the metadata offers the additional possibilities to specify validation rules within the spreadsheet itself to be checked during the entering process and not only afterwards. As we wanted to maintain the flexibility to edit the spreadsheet in different spreadsheet editors in a consistent way, this is not implemented in the dmdScheme or emeScheme [@Krug2019b], but could be easily done in other schemes.

Validate metadata and create report

To be able to validate the metadata, the validation needs access to the metadata as well as (in most cases) the data files themselves, as the validation can include checking for complete definition of the column names in tables, as implemented in the @Krug2019b. Consequentially, the metadata as well as the data needs to be uploaded to the app, which is done via the “Upload spreadsheet containing metadata” and the “Upload datafiles” buttons. In the case of the web app, these are uploaded to the server running the app (in this case https://www.shinyapps.io, which is run by RStudio Inc.).

If there are e.g. confidentiality or size reasons why an upload to a server is not an option, one could host an inhouse shiny server, which would run the shiny server and the app. The other option would be to use the local app, in which case the metadata and data remain on the local computer. Nevertheless, the data is copied into a different directory. If for example size constraints would prohibit that approach, one can use the R prompt. In this case, the validation function does not need to do any copying of the data, and only needs the path to the directory in which the data resides.

After completion of the validation, the app (web or local) downloads a report, in the default setting an html report. Optional, the report could be a word `docx` document or a `pdf`.

The report (see Figure 4) does show **errors** (which normally should be fixed), **warnings**, which are not as critical as errors and should be assessed one by one, and **notes**. The level of details, and aspects validated, in this report depends on the validation function, which can be changed in the accompanying R package.

The editing - validation cycle should be repeated until the report is satisfactory.

On the R prompt, the validation involve the two commands `validate()` and `report()` to validate the spreadsheet and to create the report from the object resulting from the validation.

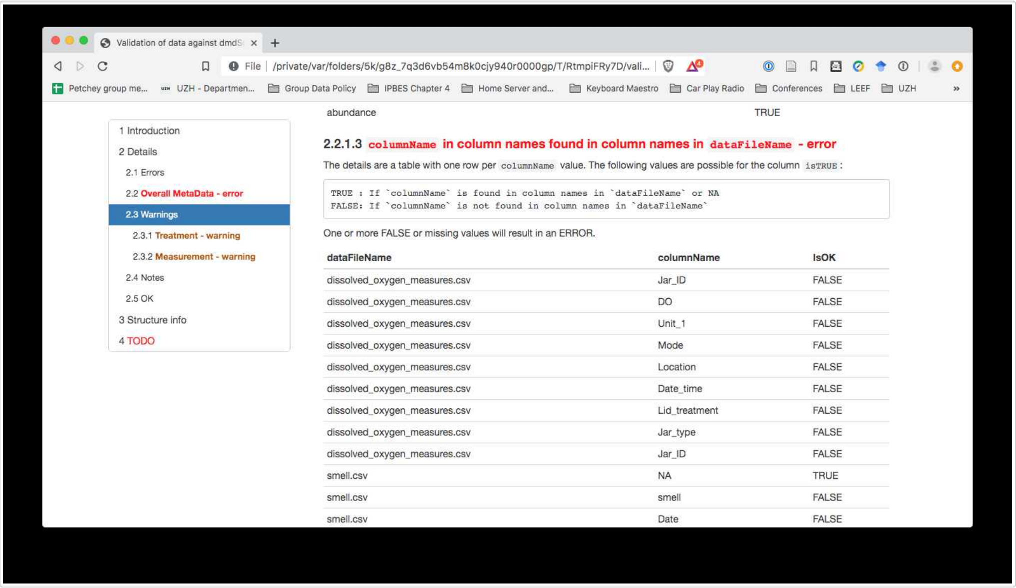

Figure 4: Example validation report, as cfrom the validation included in the `emeScheme` package [@Krug2019b].

Export uploaded spreadsheet to xml

In the app, the xml can be obtained by using the “Export to xml” button. Depending on the export functionality implemented for the selected scheme (as defined in the accompanying R package), the file returned is an `.xml` file (`dmdScheme`) or a compressed archive (`.tar.gz`) containing multiple `.xml` files (e.g. for the `emeScheme`, one per data file). On the R prompt, the command `write_xml()` will export to a single (`dmdScheme`) or multiple `.xml` files (`emeScheme`) and return the names(s) of the file(s) exported.

The R Package

The package `dmdScheme` is a base package for the usage and development of domain specific metadata schemes. It provides functionality to enter the metadata, validate the entered metadata, and export it to xml format for further processing by e.g. archival repositories. This document will give an overview over the `dmdScheme` package and what it contains.

Installation

The recommended way is to install from CRAN the stable released version:

```
install.packages("dmdScheme")
```

To install the **master** branch, the stable branch which will become the new CRAN release, from the dmdScheme repository on github, run

```
## install the devtools package if not installed yet
# install.packages("devtools")

devtools::install_github("Exp-Micro-Ecol-Hub/dmdScheme", ref = "master", build_opts = NULL)
```

If you are feeling adventurous and can live with non-working features, you can install the **dev** branch. This branch is **not stable** and features and functionality can appear or be removed without prior notice. This is, unless there is a **really** good reason, not recommended for production use:

```
## install the devtools package if not installed yet
# install.packages("devtools")

devtools::install_github("Exp-Micro-Ecol-Hub/dmdScheme", ref = "dev", build_opts = NULL)
```

Other branches are not generally recommended for installation unless you are involved in `dmdScheme` package development.

Loading the package

When you load the package, the definition of the scheme is downloaded from the dmdScheme repository installed to a temporary scheme library in a temporary directory for usage in this R session. As this scheme library is stored in a temporary directory, it will be deleted when you quit your R session and it will be re-downloaded each time you start a new session and load the package. To create a permanent package library you have to create a cache in the user directory. To do this, run

```
cache(createPermanent = TRUE)
```

and restart your R session. Now the definitions of the installed `dmdSchemes` will be installed in this user cache and be available permanently. For further info, see the documentation of the command `cache()`.

Managing installed schemes

Once the package is loaded, the default scheme definition from the package is installed and used.

There are several commands to manage installed schemes. All these commands start with `scheme_`.

These are in detail:

`scheme_default()`

returns the default scheme and version, i.e. the one with which the dmdScheme package is based. This can be the `dmdScheme` package itself, or a package created with `make_new_package()`.

```
scheme_default()
#>         name version
#> 1 dmdScheme   0.9.9
```

`scheme_repo()`

Get or set scheme repository. If `repo` is specified, the scheme repository to be used is set. Otherwise, the scheme repository used is only returned.

```
scheme_repo()
#> [1] "https://github.com/Exp-Micro-Ecol-Hub/dmdSchemeRepository/raw/master/"
```

`scheme_list_in_repo()`

Show all schemes and version available in a repo. The defaut repo is . The function reads and simply returns the file `SCHEME_DEFINITIONS.yaml` in the folder `schemes` in the repository.

```
scheme_list_in_repo()
#> $dmdScheme_0.9.5
#> $dmdScheme_0.9.5$name
#> [1] "dmdScheme"
#>
....
```

`scheme_download()`

Download a scheme definition from the repo set and stores in the destfile. The function returns the fully qualified file name to the downloaded file invisibly.

```
scheme_download(name = "emeScheme", version = "0.9.5", destfile = tempfile())
#> Warning in normalizePath(destfile): path[1]="/var/folders/50/
#> wcr5bjwn75q595n6x82gxj280000gq/T//RtmpkJVQI0/fileb21c1f7d05b6": No such file or
#> directory
```

`scheme_install()`

Install a new scheme definition. In the normal usecase, this function uses `scheme_download()` to download the scheme definition from the default [github repository](#) and installs it. The usage is

```
scheme_install(name = "emeScheme", version = "0.9.5")
#> Scheme definition `/var/folders/50/wcr5bjwn75q595n6x82gxj280000gq/T//RtmpkJVQI0/fileb21c2245f092/emeSc
#> name:     emeScheme
#> version: 0.9.5
```

`scheme_install_r_package()`

Installs the accompanying R package of the same name as the scheme. This does only install the package - it still needs to be loaded to be used!

```
scheme_install_r_package(name = "emeScheme", version = "0.9.5")
#> Package emeScheme is already installed!
#> To re-install or update, please specify `reinstall = TRUE`
```

`scheme_list()`

Shows the installed schemes.

```
scheme_list()
#>        name version
#> 1 dmdScheme   0.9.9
#> 2 emeScheme   0.9.5
```

`scheme_use()`

Activate the scheme `NAME` with version `VERSION`

```
scheme_use(name = "emeScheme", version = "0.9.5")
#> Theme switched to emeScheme_0.9.5
```

`scheme_active()`

Shows the currently active scheme.

```
scheme_active()
#>        name version
#> 1 emeScheme   0.9.5
scheme_use(name = "dmdScheme", version = "0.9.9")
#> Theme switched to dmdScheme_0.9.9
scheme_active()
#>        name version
#> 1 dmdScheme   0.9.9
```

`scheme_path_index_template()`

Returns the path to the index template. The search order is:

1. the index file included in the scheme package
2. the scheme package included in the `dmdScheme` package (**not** the acompanying package!).

```
scheme_path_index_template()
#> [1] "/var/folders/50/wcr5bjwn75q595n6x82gxj280000gq/T//RtmpkJVQI0/dmdScheme_b21c3b1dd2ee/installedSche
```

`scheme_path_xlsx()`

Returns the path to the `xlsx` file included in the scheme package.

```
scheme_path_xlsx()
#> [1] "/var/folders/50/wcr5bjwn75q595n6x82gxj280000gq/T//RtmpkJVQI0/dmdScheme_b21c3b1dd2ee/installedSche
```

`scheme_path_xml()`

Returns the path to the `xml` file included in the scheme package.

```
scheme_path_xml()
#> [1] "/var/folders/50/wcr5bjwn75q595n6x82gxj280000gq/T//RtmpkJVQI0/dmdScheme_b21c3b1dd2ee/installedSche
```

`scheme_uninstall()`

Uninstall an installed scheme. The scheme definition, is deleted from the scheme library, and moved to a temporary flder which wil be deleted at the end of the R session.

```
scheme_uninstall(name = "emeScheme", version = "0.9.5")
#> Scheme emeScheme_0.9.5 deleted and moved to
#> [1] "/var/folders/50/wcr5bjwn75q595n6x82gxj280000gq/T//RtmpkJVQI0/fileb21c4d56df46"
```

`scheme_installed()`

Return `TRUE`, if the scheme is installed

```
scheme_installed(name = "emeScheme", version = "0.9.5")
#> [1] FALSE
```

`scheme_make()`

This function is used to package a new scheme.

Entering new Metadata

To enter new data to the dmdScheme, you have to run the command

```
open_new_spreadsheet()
```

This will open Excel and the file should look similar to this, when looking at the second tab as in Figure 3.

The following points are important to remember:

1. The file is saved in a **temporary** directory. It needs to be saved at a different location, if you want to keep the changes.
2. Data can only be entered in the green cells with. All other cells are write protected.

After entering the data, save it to a location for further processing.

Importing Data from Excel Sheet

Next, you have to import the data entered in the Excel sheet into R. For simplicity, we use here a file included in the package. If you want to load your own file, replace `scheme_path_xlsx()` with the file name and path to that file.

```
x <- read_excel(
      file = scheme_path_xlsx(),
      verbose = TRUE
)
#> Transposing MdBibliometric...
#> Processing propertySet : MdBibliometric
#> Set names...
#> Set attributes...
#> Apply types...
#>    Apply type 'character' to 'uploadType'...
#>    Apply type 'character' to 'doi'...
#>    Apply type 'character' to 'publicationDate'...
#>    Apply type 'character' to 'title'...
#>    Apply type 'character' to 'description'...
#>    Apply type 'character' to 'version'...
#>    Apply type 'character' to 'language'...
#>    Apply type 'character' to 'keywords'...
#>    Apply type 'character' to 'additionalNotes'...
#>    Apply type 'character' to 'accessRights'...
#>    Apply type 'character' to 'accessRightsInfo'...
#>    Apply type 'character' to 'license'...
#> Set class...
#> Done
#> Processing propertySet : MdAuthors
#> Set names...
#> Set attributes...
#> Apply types...
#>    Apply type 'character' to 'authorID'...
#>    Apply type 'character' to 'familyName'...
#>    Apply type 'character' to 'givenName'...
#>    Apply type 'character' to 'affiliation'...
#>    Apply type 'character' to 'orcid'...
#>    Apply type 'character' to 'role'...
#> Set class...
#> Done
```

```
#> Transposing Experiment...
#> Processing propertySet : Experiment
#> Set names...
#> Set attributes...
#> Apply types...
#>    Apply type 'character' to 'name'...
#>    Apply type 'character' to 'temperature'...
#>    Apply type 'character' to 'light'...
#>    Apply type 'character' to 'humidity'...
#>    Apply type 'character' to 'incubator'...
#>    Apply type 'character' to 'container'...
#>    Apply type 'numeric' to 'microcosmVolume'...
#>    Apply type 'character' to 'mediaType'...
#>    Apply type 'numeric' to 'mediaConcentration'...
#>    Apply type 'character' to 'cultureConditions'...
#>    Apply type 'character' to 'comunityType'...
#>    Apply type 'character' to 'mediaAdditions'...
#>    Apply type 'integer' to 'duration'...
#>    Apply type 'character' to 'comment'...
#> Set class...
#> Done
#> Processing propertySet : Genus
#> Set names...
#> Set attributes...
#> Apply types...
#>    Apply type 'character' to 'speciesID'...
#>    Apply type 'character' to 'colour'...
#>    Apply type 'character' to 'density'...
#>    Apply type 'character' to 'functionalGroup'...
#>    Apply type 'character' to 'comment'...
#> Set class...
#> Done
#> Processing propertySet : Treatments
#> Set names...
#> Set attributes...
#> Apply types...
#>    Apply type 'character' to 'treatmentID'...
#>    Apply type 'character' to 'treatmentLevelHeight'...
#>    Apply type 'character' to 'comment'...
#> Set class...
#> Done
#> Processing propertySet : Measurement
#> Set names...
#> Set attributes...
#> Apply types...
#>    Apply type 'character' to 'measurementID'...
#>    Apply type 'character' to 'variable'...
#>    Apply type 'character' to 'method'...
#>    Apply type 'character' to 'unit'...
#>    Apply type 'character' to 'object'...
#>    Apply type 'integer' to 'noOfSamplesInTimeSeries'...
#>    Apply type 'numeric' to 'samplingVolume'...
#>    Apply type 'character' to 'dataExtractionID'...
#>    Apply type 'character' to 'measuredFrom'...
#>    Apply type 'character' to 'comment'...
#> Set class...
#> Done
#> Processing propertySet : DataExtraction
#> Set names...
#> Set attributes...
#> Apply types...
#>    Apply type 'character' to 'dataExtractionID'...
#>    Apply type 'character' to 'method'...
#>    Apply type 'character' to 'parameter'...
#>    Apply type 'character' to 'value'...
#>    Apply type 'character' to 'comment'...
#> Set class...
#> Done
#> Processing propertySet : DataFileMetaData
#> Set names...
#> Set attributes...
#> Apply types...
#>    Apply type 'character' to 'dataFileName'...
```

```
#>    Apply type 'character' to 'columnName'...
#>    Apply type 'character' to 'columnData'...
#>    Apply type 'character' to 'mappingColumn'...
#>    Apply type 'character' to 'type'...
#>    Apply type 'character' to 'description'...
#>    Apply type 'character' to 'comment'...
#> Set class...
#> Done
```

The `verbose = TRUE` argument will produce messages which will show you what is happening and will help to identify problems.

Print dmdScheme Data

`dmdScheme` Data can be printed by using the `print()` function. The function has three arguments which control the printout:

- `printAttr`: if `TRUE` (default) print the **basic** attributes prefixed with `A`
- `printExAttr`: if `TRUE` print the **all** attributes prefixed with `X`
- `printData`: if `TRUE` (default) print the data prefixed with `D`

```
print(
  x,
  printAttr = FALSE,
  printExtAttr = FALSE,
  printData = FALSE
)
#>  dmdScheme - dmdSchemeSet
#>      MdBibliometric - dmdSchemeData
#>      MdAuthors - dmdSchemeData
#>      Experiment - dmdSchemeData
....
```

```
print(
  x,
  printAttr = TRUE,
  printExtAttr = FALSE,
  printData = FALSE
)
#>  dmdScheme - dmdSchemeSet
#>      MdBibliometric - dmdSchemeData
#> A        Names : uploadType | doi | publicationDate | title | description | version | language | keywor
#> A        Units :
....
```

```
print(
  x,
  printAttr = TRUE,
  printExtAttr = TRUE,
  printData = FALSE
)
#>  dmdScheme - dmdSchemeSet
#>      MdBibliometric - dmdSchemeData
#> A        Names : uploadType | doi | publicationDate | title | description | version | language | keywor
#> A        Units :
....
```

```
print(
  x,
  printAttr = TRUE,
  printExtAttr = TRUE,
  printData = TRUE
)
#>  dmdScheme - dmdSchemeSet
#>      MdBibliometric - dmdSchemeData
#> A       Names : uploadType | doi | publicationDate | title | description | version | language | keywor
#> A       Units :
....
```

Validating your metadata

The metadata in the spreadsheet can be validated by using the following command and which results in an object of class `dmdScheme_validation`.

```
validate( scheme_path_xlsx() )
#> Validating Experiment
#> Validating MdBibliometric
#> Validating MdAuthors
#> Validating Genus
#> Validating Treatments
#> Validating Measurement
#> Validating DataExtraction
#> Validating DataFileMetaData
#> $error
#> [1] 3
#>
#> $details
....
```

To create a report (html, pdf or docx) yo can use the `report()` function:

```
report( scheme_path_xlsx() )
```

which will open a html report in your browser.

Converting Metadata to xml

You can export the data to an an `xml_document` object as defined in the `xml2` package to do further processing:

```
x <- read_excel( scheme_path_xlsx() )
xml <- as_xml( x, file = xmlFile)
xml
#> {xml_document}
#> <dmdScheme fileName="dmdScheme_0.9.9.xlsx" dmdSchemeName="dmdScheme" dmdSchemeVersion="0.9.9" property
#> [1] <MdBibliometricList>\n  <MdBibliometric>\n    <uploadType>Dataset</upload ...
#> [2] <MdAuthorsList>\n  <MdAuthors authorID="1">\n    <authorID>1</authorID>\n ...
....
```

You can save the xml to a file by using

```
xmlFile <-  tempfile(fileext = ".xml")

xml2::write_xml(x = xml, file = xmlFile)
#> NULL
```

or directly in one command

```
xmlFile <-  tempfile(fileext = ".xml")

write_xml( x = x, file = xmlFile )
#> [1] "/var/folders/50/wcr5bjwn75q595n6x82gxj280000gq/T//RtmpkJVQI0/fileb21c63e67aed.xml"
```

Re-import from xml into R

```
x <- read_xml( xmlFile )
x
#>  dmdScheme - dmdSchemeSet
#>      MdBibliometric - dmdSchemeData
#> A       Names : uploadType | doi | publicationDate | title | description | version | language | keywor
#> A       Units :
....
```

Developing new dmdSchemes derived scheme definitions

This package contains all the functionality to easily create a new metadata scheme. The resulting scheme package will contain all files needed so that it can be easily be distributed and used, and can be either be distributed directly or uploaded to an online repository, e.g. the default dmdScheme repository.

A new scheme definition only has to include the scheme definition and example data. It can also contain additional examples and an R script to install the accompanying R package, but these are not required.

Create and edit new definition

To create the scheme itself (as shown in Figure 5), it is recommended to begin with an existing scheme. It is possible to edit most aspects in the scheme definition `xlsx` file, including adding or deleting tabs (only the Experiment and DataFileProperties tabs are needed), adding or removing rows ("vertical" tabs like e.g. the Experiment tab) or columns (horizontal (other) tabs), changing the types, suggested values, allowed values and units. **If the `.xlsx` file is locked, you can unlock it by using the password `test`.**

It is important that the final scheme contains example data as - internal processing of the `.xlsx` file require example data and will fail if a scheme without example data is provided, and - it is much more user friendly if the user of a package not only sees the empty scheme, but also a filled in scheme with example data.

This `.xlsx` file can be edited following the limitations as specified below. After modifications have been done and the spreadsheet has been saved, the scheme package can be created by using

```
scheme_make( "TheFile.xlsx" )
```

which will create a file named `NAME_VERSION.tar.gz` in the directory `path` based on the scheme definition in `TheFile.xlsx`. `NAME` and `VERSION` are the scheme name and scheme version as specified in the cell `(H:1)` in sheet `Experiment`.

The following two commands will install the new scheme and use it:

```
scheme_install(file = "NAME_VERSION.tar.gz")
scheme_use("NAME", "VERSION")
```

To upload the scheme to the main repository on github, please either clone the repo and send a pull request or file an issue with the scheme package as an attachment.

Minimum requirements for metadata schemes derived from `dmdScheme`

There are a few minimum requirements for `dmdScheme` derived metadata schemes, so that all functions in the `dmdScheme` package will work.

These are:

1. Tab name has to be identical to `propertySet` value (Cell `A:2` in the tab Experiment, `B:1` in other tabs)
2. The tab named `Experiment` is required. This tab is different to the others as it has
    1. horizontal layout

2. only one value per property allowed
3. the top-right cell, i.e. the one above the values to be entered, contains the word **DATA**, **name of the scheme** and **version of the scheme** separated by a space, e.g. **DATA emeScheme v0.9.5**

3. The tabs `MdBibliometric` and `MdAuthors` are required as they contain all the bibliometric and author metadata
4. The tab named `DataFileMetaData` is required
   - one column named `dataFileName`
5. Each tab except `Experiment`, `DataFileMetaData` and `MdBibliometric` needs an ID field.
   - The first column in the tab must be a column named `...ID`.
6. The spreadsheet can contain a tab `DOCUMENTATION`. This tab can contain information for the user and will not be imported.

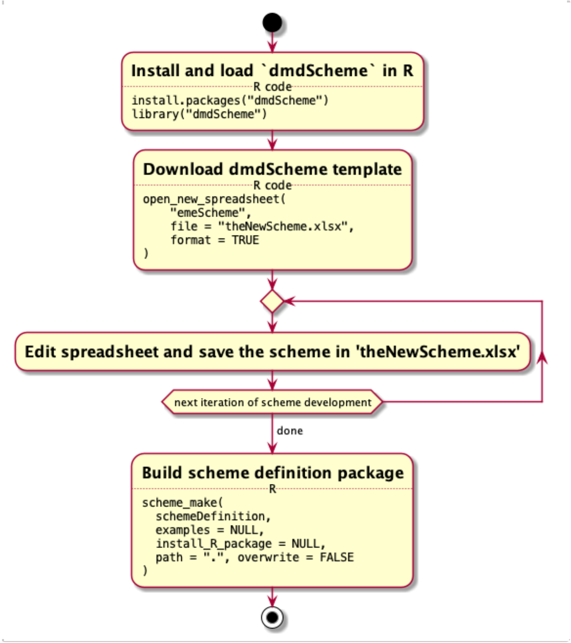

Figure 5: Create a new domain specific scheme based on dmdScheme.

Developing accompanying R packages

The package `dmdScheme` package is only providing the base functionality for working with dmdSchemes. In many cases, the functionality needs to be extended to be able to work with other schemes. This can be done by creating an accompanying R package. It is a standard R package which has to

- depend on the `dmdScheme` package (as it extends it's capabilities), and
- automatically downloads, installs and activates a specific scheme definition and not the default dmdScheme.

It is not necessary to build an accompanying R package, but doing so will make it possible to adapt many aspects of the validation and export process to the needs of a specific domain.

The easiest method to develop an accompanying R package is to - use `scheme_use()` to activate a new scheme for which the accompanying package should be developed - use the convenience function `make_new_package()` to create a package skeleton which fulfils these requirements

Now, functions can be added. For an example see https://github.com/Exp-Micro-Ecol-Hub/emeScheme, in which the methods `as_xml_list.emeSchemeSet.R` and `validate.emeSchemeSet_raw.R` extend the generic functions `as_xml_list()` and `validate()`.
