# Supplementary material for: Metadata Made Easy: Develop and Use Domain‐Specific Metadata Schemes by following the dmdScheme approach
Source: Ecol Evol. 2021 Jun 25;11(14):9174–81. doi: 10.1002/ece3.7764 (PMC8293710; doi:10.1002/ece3.7764)
Supplement: Supplementary file 3 — Supplementary Material [file ECE3-11-9174-s003.pdf]

User Manual for Experimental Microbial Ecology Metadata tool

Owen L. Petchey and Rainer M. Krug

2021-01-12

- Introduction
- What is *useful* metadata?
- What metadata is covered in the emeScheme, and what is not
- At what point of my study should I enter metadata
- Getting started
  - Working with the universal web app
  - Working programatically via R
- Preparing data for archiving
  - About dates and times
  - A worked example of preparation of data for archiving
- Fill in the Metadata
  - MdBibliometric worksheet
  - MdAuthors worksheet
  - Experiment worksheet
  - Species worksheet
  - Treatments worksheet
  - Measurements worksheet
  - DataExtraction worksheet
  - DataFileMetaData worksheet
- Validation
- Fixing issues
- Making the final xml version
- Depositing in a repository

Introduction

Researchers are increasingly required to make the data they produce **F**indable, **A**ccessible, **I**nteroperable, and **R**eusable (wikipedia FAIR entry). Providing metadata that describes studies, experiments, treatments, measurements, data, and so on is a key part of this endeavour.

The aim of the experimental microbial ecology metadata tool (`emeScheme` R package on https://github.com/Exp-Micro-Ecol-Hub/emeScheme) and the associated experimental microbial ecology experiments metadata scheme (`emeScheme` scheme package on https://github.com/Exp-Micro-Ecol-Hub/dmdSchemeRepository) is to help you describe your study and its data, i.e. to produce useful metadata. You will do this by:

1. Preparing your datasets for archiving (see Prepare Data for Archiving),
2. Entering your metadata into the spreadsheet supplied (see Fill in Metadata),
3. Validating the combination of the metadata and data (this will be largely automatic) (see Validation),
4. Fixing any issues revealed by the validation step, until satisfactorily validated (see Fixing issues).
5. Deposit the metadata and data in a repository of your choice (see Depositing in a repository).

What is *useful* metadata?

Imagine we would like to use the metadata to find experiments (and potentially return the relevant data from them) that involve *Colpidium striatum* grown at **20ºC** in **0.55g per litre protist pellet medium**. To do this we need to ensure that the **species name**, the **temperature**, and the **growth media information** are contained in the metadata. We also need to make sure that when temperature is described in a **standard fashion**, and that the growth media description is also standardised. So there must be some standardisation. But its clear that most experiments have some unique features, such that we can't standardise everything in advance. The metadata scheme we use here is intended to be a good balance of standardised and flexible.

What metadata is covered in the emeScheme, and what is not

The emeScheme metadata only covers what is necessary/helpful to describe an "experimental microbial ecology" study (though as an aside we think/wonder if it might be useful for many other settings of ecological experiments). Some important/essential metadata that is also required by other schemes, such as the *DataCite* scheme and/or the *Ecological Metadata Language/Scheme*, are included (e.g. some bibliographic information)

but much is not. We may add tools that help researchers enter other metadata and to export to other schemes.

At what point of my study should I enter metadata

You will benefit from starting metadata entry when you design your study. You'll likely find out that doing so will help with the planning of the study. You may then choose to work in a slightly different way, since this will make things more efficient later (this might especially be so for data handling issues). No problem, however, if you decide to do it at the end (or you are reading this after a study is complete).

Getting started

There are three different ways of using the **emeScheme**. You can either use it as a web-app at https://rmkrug.shinyapps.io/dmd_app/, as a web-app locally, or from the R prompt programatically. A comparison of these three methods can be found in the "Develop and use the `dmdScheme`" vignette in the **dmdScheme** package.

Working with the universal web app

The web app can be started as a shiny app. Most of the app is self explanatory, and the workflow consists of

1. activating the scheme from the list of available schemes. This list is auto-populated from the scheme repository. Consequently, it will always contain the newest schemes. Activating a scheme will also install and load the accompanying R package.
2. Download an Excel sheet which is used to enter the metadata (see details below). It is also possible to download an excel sheet with example data.
3. The Excel sheet with the entered metadata is uploaded to the server for further processing.
4. Additional data files, when needed for the validation, can also uploaded
5. The validation can be done and the validation report can be downloaded as a html (the best option), pdf or docx file.
6. Finally, the metadata can be exported to one (or more) xml files. How these do exactly look, depends on the scheme and the in the accompanying R package implemented export functions.

The web app can also be run locally, by running the command `run_app()` after loading the package `dmdScheme`.

Further details can be found in the next section.

Working programatically via R

The first thing to do is get and load the **emeScheme** R package. This includes to install and load the **dmdScheme** package from CRAN and than install the **emeScheme** scheme package and the accompanying R package.

You can install the base package **dmdScheme** from CRAN or from github. We recommend instaling from CRAN, as this is the officially supported version. The master branch on masterbranch on github is usually also stable, but the dev branch contains the development version and should be avoided for normal usage. To install the package, you do

```
## install the `dmdScheme` package fron CRAN

install.packages("dmdScheme")

## you can also install it from github, for which you need the `devtools` package

# install.packages("devtools")
# devtools::install_github("Exp-Micro-Ecol-Hub/dmdScheme", ref = "master", build_opts = c("--no-resave-dc
```

Now you have to install the **emeScheme** scheme package. This scheme package, which can be installed from the scheme repository, contains the scheme definition, examples, and a script to install an R package which enhances the functionality. The scheme is installed into a temporary library which is deleted when you quit R, while the R package is installed in the normal R library.

You have to 1. load the package **dmdScheme**, 2. download and install the scheme definition for the `emeScheme` in a temporary scheme library by running `scheme_install()`, and the acompanying R package (which has the same name as the scheme package, i.e. `emeScheme`)

```
library("dmdScheme")

scheme_install( name = "emeScheme", version = "0.9.9", install_package = TRUE)
```

3. load the accompanying package **emeScheme**

```
library(emeScheme)
```

The scheme needs to be installed each time you load the package in a new R session, as the session library is only temporary, but the accompanying R package is installed permanently.

To make the scheme library permanent, run

```
cache(createPermanent = TRUE)
```

and restart your R session. This will create a permanent cache which is used for the scheme library in your user directory, so that schemes are installed permanently.

For details on the managing of schemes, see the vignette “Develop and Use the dmdScheme” in the **dmdScheme** package.

Even without a permanent scheme library, you can the next time simply use

```
library("emeScheme")
```

to load the R package and, when needed, install the scheme package.

Now you can start entering metadata.

To enter the metadata run this R command to get an Excel version of the emeScheme without metadata in it:

```
open_new_spreadsheet()
```

If you are told there are problems with opening the spreadsheet then try to repair it automatically. If this does not work, use `open_new_spreadsheet(format = FALSE)`

The following command opens the sheet with the example data in:

```
open_new_spreadsheet(keepData = TRUE)
# open_new_spreadsheet(keepData = TRUE, format = FALSE)
```

Either way, make a copy of the spreadsheet and save it somewhere safe, like in the folder for your study/experiment.

Its probably worth now looking at the spreadsheet (but don’t yet try to fill in anything), to see what metadata you will need to enter. There are several worksheets:

- **MdBibliometric** Bibliometric data about the data package (and not about any associated research papers!).
- **MdAuthor** Information uch as ORCID and role for each person involved in the creation and preparation of the data package.
- **Experiment** Here you enter details of your experiment, e.g. what temperature was the experiment conducted at. If temperature was a treatment, then you state that here and supply the levels in the **Treatments** worksheet.
- **Species** Give details of the species involved in the experiment. If the species are unknow, enter `unknown`,
- **Treatments** Here we give the manipulations involved in the experiment.
- **Measurements** Details of what was measured, and how data was extracted from the measurements.
- **DataExtraction** Details of how measurements were extracted from raw data, if that happened.
- **DataFileMetaData** Details of the data files, description of the variables they contain, and validation conditions for the data.

Preparing data for archiving

There are no hard and fast rules, but there are some guidelines that will make things easier, or are otherwise recommended for datasets (e.g. data that we can have in a spreadsheet):

- Use long / tidy arrangement of data.
- Use a separate variable for each treatment.
- Always include a variable that allows data file to be related to others (e.g. Microcosm_ID).
- Do not put different measurement types in a single data file. E.g. do not put measures of dissolved oxygen measures and abundance data in the same data file.

If your data does not fit these guidelines, e.g. is a collection of videos files or a collection of files containing DNA sequence data, create a summary table which lists the files and gives a description on the treatment from which the files resulted. In this way, your datafile is the summary table, and your individual file (video, image, DNA sequence file, …) is one datapoint. You can either add all these in an additional directory or put them into one container of some kind (e.g. compressed folder (tar.gz preferably) or zip file).

About dates and times

It will be very useful if during data preparation you standardise any dates and times in the data. A good method for this is to use functions in the **lubridate** R package, such as `dmy`, `hms`, and `dmy_hms` to create the standardise dates, and then write these to the ready for research/archiving data version.

A worked example of preparation of data for archiving

The package `emeScheme` includes examples of raw data, processing scripts, processed data for archiving and the associated metadata.

The examples (so far only two) are: - `basic`: a basic simple example - `ex_2`: an example of more complex metadata

You can always see a list of examples included in the active scheme by running the command

```
make_example()
```

```
## Included examples are:
```

```
## basic
## ex_2
```

You can get one of the examples, e.g. the basic example by running:

```
make_example("basic")
```

This will copy the basic example into your current working directory in a folder named `basic`.

The worked **basic** example shows appropriate preparation of data for archiving and ease of further use. Its an example of what you could do, not what you have to do, though probably you benefit from doing something similar.

The `basic` folder contains:

- An R-project file `expt1.Rproj`
- A spreadsheet file `emeScheme.xlsx`
- A folder named `data` with two subfolders:
    - A `raw_data` folder containing files and folders of files that contain the data as it was entered by the researcher, or produced by the measurement machine.
    - A `archiving_data` folder containing data files that have been prepared for archiving.
- A `code` folder containing two files:
    - `data_preparation.Rmd` containing code that takes the information in the files in the `raw_data` folder, prepares this information for easy use by researchers (which also makes them suitable for archiving), and then writes this nicely prepared information/data into files in the `archiving_data` folder.
    - `analyses.Rmd` starts by reading the nicely prepared data files from the `archiving_data` folder, and would then proceed with analyses.

You will not be able to use the code in `data_preparation.Rmd` outside this example as it is highly specific to this example.

Note that it is **very important** that the preparation of the data for easy use by researchers and for archiving (done in `data_preparation.Rmd`) is completely transparent.

If you want to try it, delete all the files in the `data/archiving_data` folder and knit the `data_preparation.Rmd` or execute / source the `data_preparation.R` file which will re-create the files in the `archiving_data` folder.

Now we have nicely prepared data. We are ready to fill in the metadata.

Fill in the Metadata

There's no more putting it off… lets try filling in the metadata. First, find and open your copy of the metadata spreadsheet you made.

If you're working through the worked example (`basic`), open the `emeScheme.xlsx` file.

- Green cells are where you enter information.
- Pink cells are places where you should not try to type anything (they should be locked anyway).

Two important points to remember: 1. **Please don't not use any formulas! Only enter values directly! Formulas can (and likely will) cause strange error messages later on!** 2. **Put an entry in all cells: enter "NA" in cells which do not have a value for your data set. This avoids misunderstandings. The `comment` column can be left empty if you don't have any additional remarks.**

MdBibliometric worksheet

This worksheet (together with MdAuthor) contains the bibliometric metadata, i.e. the metadata related to authorship, licenses, keywords, etc. The fields are self explanatory.

MdAuthors worksheet

This sheet contains everybody involved to such a degree in the measurement, curation and other aspects involving the data gathering itself as well as the creation of this data package (datasets + metadata + other accompanying documents), which warrants the inclusion in the list of "authors" of the data package.

Every scientist who publishes, should be encouraged to get a unique ID. One of the currently most widely used is the ORCID ID. This ID should be entered in the column `orcid`. The last column contains the role or roles each person has played in this process. The role is defined by CRediT system, the Contributor Roles Taxonomy. One author can have more than one role.

Experiment worksheet

This one should not need too much explanation.

One important point is, however, what to enter in a field (e.g. the temperature field) if your experiment involved different temperatures (i.e. if temperature was a treatment). The answer is you should enter the word "treatment" as the value of this field, to indicate that temperature was a treatment in your experiment.

When you fill in this information, think about standardisation. If you enter a temperature, you should therefore enter only a numeric value in degrees Celcius. Writing "twelve degrees" is going to be quite unhelpful! (We could constrain the possible entries here, but then we lose flexibility; recall the discussion above about balancing standardisation and flexibility.)

Something else of note: Look at the field `mediaAdditions` and you will see the entry is "Wheat seeds added on specific dates, see file wheat_seed_additions.csv". Here we see the possibility for great flexibility… we refer to information in a separate data file. Note though that this data file is just a list of dates and number of wheat seeds added – it is not information about an experimental treatment, because all experimental treatments experience the same additions (i.e. these additions are feature of the experiment). In addition, the information in these additional files is **not indexed** when submitting the data to a repository for archiving. In other words: researcher will be able to search for all entries in this metadata sheet, but not be able to directly search the data in the data files (data files, additional files as wheat_seet_additions.csv)! So such information as is in the wheat_seed_additions.csv file are effectively hidden from such searches. This is an example of flexibility dominating over standardisation, with some findability thereby being lost.

If you believe that other features of your experiment are important to include, please do so by adding these in the `comment` row. If they seem to be not appropriate to add there, you may need to think about extending the emeScheme.

Species worksheet

This worksheet should also not need much explanation. Note that this is just a list of the species in the experiment. Its not a description of any treatments involving species composition. These have to be specified in the `Treatment` worksheet.

During validation of your metadata the species names will be automatically checked against online databases of species names, and you will get a report on matches or lack of matches. (This helps increase interoperability of the data.)

In the worked example the experiment was set up by adding a pool of unknown species composition (*Tetrahymena thermophila* only added for illustrative purposes). So no entries in this worksheet. The composition could be "measured" using e.g. molecular methods, and would then be an entry in the **Measurements** worksheet (see below for more details on that worksheet).

If any of your data sets contain species names, it will be very helpful to make them match the names in this sheet. You will be told about the presence or absence of such matches in the validation report.

Treatments worksheet

Treatments worksheet

Here enter in each row each of the treatment levels in each treatment. In the “parameter” column put the name of the treatment (in the worked example this is “Lid_treatment”).

You do not need to, and should not, attempt to include information about treatment combinations. So if your experiment involved a temperature treatment with three levels (15, 20, and 25) and a species composition treatment with four treatments (species A alone, species B alone, species C alone, and all three together) you would enter seven rows of information. You would not enter 12 rows if your experiment was a two-way fully factorial design with these two treatments.

It would be very nice here (i.e. it will make your life easier) to make the names of your treatment and the descriptions of the levels exactly the same as those used in the related columns in your data files. You will be told about the presence or absence of such matches in the validation report.

As species composition can be seen as a form of treatment, they have to be entered here. In this case, the `unit` should be **speciesID** and the `treatmentLevel` should contain a comma separated list of `speciesID`s.

Measurements worksheet

Here enter a row of information for each type of measurement made. In the worked example there are four types of measurement: oxygen concentration, community composition, smell, and DNA sequences. Much of the information required in the rows should be quite self explanatory, apart perhaps from:

- `dataExtractionID`: Fill in something here if the measurement required some processing of another data file / measurement. E.g. in the worked example the commmunity composition measure (**abundance**) comes from the **sequenceData** data (`measuredFrom` coumn) by extraction method **Mol_Analy_pipeline1** (see DataExtraction for further details).
- `measuredFrom`: should be one of the names of the other rows of measurements. E.g. in the worked example the community composition measure comes from the **sequenceData** measurement, or `raw` if it is raw data directly from the measurements.

It would be **very** nice/useful (i.e. make your life easier) if the entries in the `measurementID` column correspond with the names of the columns in the data file containing the data (see DataFileMetaData for further details). You will be told about the presence or absence of such matches in the validation report.

DataExtraction worksheet

Here you can give details of any (and please all - this will be validated in the validation report) data extraction methods mentioned in the **Measurements** worksheet. You can enter any parameters and values of these you like (i.e. there is high flexibility here because we do not anticipate a lot of standardisation to exploit). In the worked example we wrote “See description in file xxx.yyy”. This file could be a script or text description of the pipeline.

DataFileMetaData worksheet

Here you give a row for each of the variables in each of the data files.

- **dataFileName** Here enter the file name of the data file.
- **columnName** Here enter the name used in the datasheet / database / tabular data. This can be the exact name, a regular expression, or an expression with wildcards (`*` and `?`). If wildcards has used, they need to be enclosed in triple exclamation marks, i.e. `Species_!!!*!!!` would match `Species_1` as well as `Species_123`. If the *columnData mappingColumn* combination refers to a condition for the whole data file, e.g. when only one species combination is used in the experiment from this data set, than enter *NA*.
- **columnData** Here enter whether the variable/column in the datafile contains a *Measurement*, a *Treatment*, an *ID* variable, *Species* or something else (*other*).
- **mappingColumn** If
  - **columnData = Measurement**: enter the *measurementID* as entered in the *Measurement* sheet,
  - **columnData = Treatment**: enter the *treatmentID* as entered in the *Treatment* sheet,
  - **columnData = Species**: enter the *treatmentID* as entered in the *Treatment* sheet which refers to the species composition as `treatmentLevel`, or *NA*.
- **type** Enter the variable type (e.g. numeric, character, date).
- **description** Anything you think relevant. Please enter the date/time format for variables that contain date/times.

At the validation stage below you will get a report on things such as matches and lack of matches with entered variable names and those in the actual datasets, matches of treatment names and measurement names, absences of data about things that were measured, absence about treatment information for things that were measured, and so on.

Note that the example contains a datafile in this worksheet that does not exist in the data folder. This is intended to simulate an error (i.e. forgetting to include a datafile) and to then result notification of this error in the

validation report.

Validation

This is work in progress. Please give it a go and have a look at the produced validation report, but note that we are leaving most of the development and refinement to the next stage of development.

After entering your data, you should do a validation test of your metadata and data. The validation will check many different aspects of your metadata and data, and returns a report (html, pdf, docx or all three). The validation and resulting report is a work in progress.

To produce a validation report, use the `report` function and give it your spreadsheet, the path to your data folder, and the type of report you want:

```
report("thenameofyourmetadataspreadsheet.xlsx", path = "the/path/to/your/datafiles", report = "html")
```

As mentioned already, the validation and resulting report is a work in progress. Please let us know if you suggest particular validation checks.

Fixing issues

The validation report will suggest improvement you can make to your metadata. It may also say that some changes/additions are *required* in order to produce valid metadata. The more of these improvements you can make, the more useful will be your metadata-data combination.

Making the final xml version

The Excel version of the metadata is most useful for us to enter the metadata easily. It is not so useful for standardised/automated metadata enquiry/search/submission… i.e. Excel versions of metadata are not really [FAIR] compliant. The metadata gurus decided that it should be stored as xml files, as it is plain text, it is in principle readable by humans (though is not meant to be read by humans really), it is machine readable (and then easily converted into any other format, e.g. ones easy for humans to read).

Therefore, you should submit the final xml version of your metadata. Only do this after you have a satisfactory validation.

As the metadata as it is entered in the spreadsheet contains metadata for *many* different data files (as entered in the column `DataFileMetaData / dataFileName`), the metadata will be split and exported to multiple xml files.

```
write_xml( read_excel("thenameofyourmetadataspreadsheet.xlsx", file = "thenameofyourmetadataspreadsheet.x
```

This will create one xml file per data file specified in the DataFileMetaData tab.

If you want to save the metadata for later usage or analysis in R, you should use standard R functions like

```
saveRDS( x = read_excel("thenameofyourmetadataspreadsheet.xlsx", file = "thenameofyourmetadataspreadsheet
```

which will also save the metadata in an `rds` file.

You can read it in again by using

```
readRDS( file = "thenameofyourmetadataspreadsheet.RDS" )
```

Depositing in a repository

We suggest that your metadata (the produced xml file and the excel file), data files, and validation report are deposited as one submission to a repository such as dryad or zenodo.
