# Supplementary material for: Metadata Made Easy: Develop and Use Domain‐Specific Metadata Schemes by following the dmdScheme approach
Source: Ecol Evol. 2021 Jun 25;11(14):9174–81. doi: 10.1002/ece3.7764 (PMC8293710; doi:10.1002/ece3.7764)
Supplement: Supplementary file 4 — Supplementary Material [file ECE3-11-9174-s004.pdf]

1 Introduction

2 Details

3 Structure info

4 TODO

Validation of data against dmdScheme

Tester

2020-04-16

- **Author**: Tester
- **dmdScheme metadata name**:
- **data package path**: `add this`

1 Introduction

This validation report validates the most likely an excelsheet containing the data. The headers do represent sections of validations, while the bullets reperesent individual validations. Sections of the validation and are hierarchical, i.e. a higher level validation result depends on the lower level validation results. The headers and validations follow the structure of

Error_Level : **Name of the validation**

The error level can be: - **OK**: the validation passed - **note**: the validation contains an inconsistency which migh be an error or on purpose. Please check these! - **warning**: the validation contains an inconsistency which could be an error or on purpose. Please check these! - **error**: the validation failed and contains an error - needs to be fixewd before the data can be exported!

An ideal validation will not contain any errors or warnings.

Validation results are accumulated by forwarding the highest error to the validation of the next level. If validation 1.3. contains an error, 1. will be classified as an error as well, irrespective of 1.1, 1.2, ….

The details do contain the individual tests and are explained in detail in the sections

Details **Error_Level** : **Name of the validation**

2 Details

```
valErr_extract(result) %>%
  table %>%
  set_names(valErr_info(names(.))$text)
```

```
##    OK  note error
##    12     4    11
```

2.1 Errors

```
print(result, level = 2, listLevel = 20, type = "details", error = 3)
```

2.2 Overall MetaData - error

The details contain the different validations of the metadata as a hierarchical list. errors propagate towards the root, i.e., if the ‘worst’ is a ‘warning’ in a validation in `details` the error here will be a ‘warning’ as well.

x
NA

2.2.1 Genus - error

The details are a table with one row per unique validation. The column `Module` contains the name of the validation, The column `error` contains the actual error of the validation. The following values are possible for the column `isTRUE` :

```
TRUE : If the validation was `OK`.
FALSE: If the validation was an `error`, `warning` or `note`.
NA   : If at least one v alidation resulted in `NA
```

One or more FALSE or missing values values will result in an ERROR.

Module	errorCode	isOK
types	OK	TRUE
suggestedValues	note	FALSE
allowedValues	error	FALSE
IDField	OK	TRUE

2.2.1.1 values in allowedValues - error

The details are a table of the same dimension as the input (green) area of the meatadata sheet. The following values are possible:

```
FALSE: If the cell value is not contained in the allowedValues list.
TRUE : If the cell value is contained in the allowedValues list.
NA   : empty cell or no allowed values specified
```

One or more FALSE values will result in an ERROR.

speciesID	colour	density	functionalGroup	comment
FALSE	FALSE	FALSE	FALSE	FALSE
FALSE	FALSE	TRUE	FALSE	TRUE

2.2.2 Treatments - error

The details are a table with one row per unique validation. The column `Module` contains the name of the validation, The column `error` contains the actual error of the validation. The following values are possible for the column `isTRUE` :

```
TRUE : If the validation was `OK`.
FALSE: If the validation was an `error`, `warning` or `note`.
NA   : If at least one v alidation resulted in `NA
```

One or more FALSE or missing values values will result in an ERROR.

Module	errorCode	isOK
types	OK	TRUE
suggestedValues	note	FALSE
allowedValues	error	FALSE
IDField	OK	TRUE

2.2.2.1 values in allowedValues - error

The details are a table of the same dimension as the input (green) area of the meatadata sheet. The following values are possible:

```
FALSE: If the cell value is not contained in the allowedValues list.
TRUE : If the cell value is contained in the allowedValues list.
NA   : empty cell or no allowed values specified
```

One or more FALSE values will result in an ERROR.

treatmentID	treatmentLevelHeight	comment
FALSE	FALSE	TRUE
FALSE	FALSE	TRUE
FALSE	FALSE	TRUE
FALSE	FALSE	TRUE
FALSE	FALSE	TRUE

2.2.3 Measurement - error

The details are a table with one row per unique validation. The column `Module` contains the name of the validation, The column `error` contains the actual error of the validation. The following values are possible for the column `isTRUE` :

```
TRUE : If the validation was `OK`.
FALSE: If the validation was an `error`, `warning` or `note`.
NA   : If at least one v alidation resulted in `NA
```

One or more FALSE or missing values values will result in an ERROR.

Module	errorCode	isOK
types	OK	TRUE
suggestedValues	note	FALSE
allowedValues	error	FALSE
IDField	OK	TRUE

2.2.3.1 values in allowedValues - error

The details are a table of the same dimension as the input (green) area of the meatadata sheet. The following values are possible:

```
FALSE: If the cell value is not contained in the allowedValues list.
TRUE : If the cell value is contained in the allowedValues list.
NA   : empty cell or no allowed values specified
```

One or more FALSE values will result in an ERROR.

measurementID	variable	method	unit	object	noOfSamplesInTimeSeries	samplingVolume	dataExtractionID	measuredFrom	comment
FALSE	FALSE	FALSE	FALSE	FALSE	FALSE	FALSE	FALSE	FALSE	TRUE
FALSE	FALSE	FALSE	FALSE	FALSE	FALSE	FALSE	FALSE	FALSE	TRUE
FALSE	FALSE	FALSE	FALSE	FALSE	FALSE	FALSE	FALSE	FALSE	TRUE
FALSE	FALSE	FALSE	FALSE	FALSE	FALSE	FALSE	FALSE	FALSE	TRUE

2.2.4 DataExtraction - error

The details are a table with one row per unique validation. The column `Module` contains the name of the validation, The column `error` contains the actual error of the validation. The following values are possible for the column `isTRUE` :

```
TRUE : If the validation was `OK`.
FALSE: If the validation was an `error`, `warning` or `note`.
NA   : If at least one v alidation resulted in `NA
```

One or more FALSE or missing values values will result in an ERROR.

Module	errorCode	isOK
types	OK	TRUE
suggestedValues	note	FALSE
allowedValues	error	FALSE
IDField	OK	TRUE

2.2.4.1 values in allowedValues - error

The details are a table of the same dimension as the input (green) area of the meatadata sheet. The following values are possible:

```
FALSE: If the cell value is not contained in the allowedValues list.
TRUE : If the cell value is contained in the allowedValues list.
NA   : empty cell or no allowed values specified
```

One or more FALSE values will result in an ERROR.

dataExtractionID	method	parameter	value	comment
FALSE	TRUE	TRUE	TRUE	FALSE

2.2.5 DataFileMetaData - error

The details are a table with one row per unique validation. The column `Module` contains the name of the validation, The column `error` contains the actual error of the validation. The following values are possible for the column `isTRUE`:

```
TRUE : If the validation was `OK`.
FALSE: If the validation was an `error`, `warning` or `note`.
NA   : If at least one v alidation resulted in `NA
```

One or more FALSE or missing values values will result in an ERROR.

Module	errorCode	isOK
types	OK	TRUE
allowedValues	error	FALSE
dataFilesExist	error	FALSE

2.2.5.1 values in allowedValues - error

The details are a table of the same dimension as the input (green) area of the meatadata sheet. The following values are possible:

```
FALSE: If the cell value is not contained in the allowedValues list.
TRUE : If the cell value is contained in the allowedValues list.
NA   : empty cell or no allowed values specified
```

One or more FALSE values will result in an ERROR.

dataFileName	columnName	columnData	mappingColumn	type	description	comment
FALSE	FALSE	TRUE	TRUE	TRUE	TRUE	TRUE
FALSE	FALSE	TRUE	FALSE	TRUE	TRUE	TRUE
FALSE	FALSE	TRUE	TRUE	TRUE	TRUE	TRUE
FALSE	FALSE	TRUE	TRUE	TRUE	TRUE	TRUE
FALSE	FALSE	TRUE	TRUE	TRUE	TRUE	TRUE
FALSE	FALSE	TRUE	TRUE	TRUE	FALSE	TRUE
FALSE	FALSE	TRUE	FALSE	TRUE	TRUE	TRUE
FALSE	FALSE	TRUE	TRUE	TRUE	TRUE	TRUE
FALSE	FALSE	TRUE	TRUE	TRUE	TRUE	TRUE
FALSE	TRUE	TRUE	FALSE	TRUE	TRUE	TRUE
FALSE	FALSE	TRUE	FALSE	TRUE	TRUE	TRUE
FALSE	FALSE	TRUE	TRUE	TRUE	FALSE	TRUE
FALSE	FALSE	TRUE	FALSE	TRUE	TRUE	TRUE
FALSE	FALSE	TRUE	TRUE	TRUE	TRUE	TRUE
FALSE	TRUE	TRUE	FALSE	TRUE	TRUE	TRUE
FALSE	FALSE	TRUE	TRUE	TRUE	TRUE	TRUE
FALSE	FALSE	TRUE	TRUE	TRUE	FALSE	TRUE
FALSE	FALSE	TRUE	FALSE	TRUE	TRUE	TRUE
FALSE	FALSE	TRUE	TRUE	TRUE	TRUE	TRUE
FALSE	FALSE	TRUE	FALSE	TRUE	TRUE	TRUE

2.2.5.2 `dataFile` exists in path - error

The details are a table with one row per unique `variable` The following values are possible for the column `isTRUE`:

```
TRUE : If `dataFile` exist in the given `path`
FALSE: If `dataFile` does not exist in the given `path`
NA   : empty cell
```

One or more FALSE or missing values will result in an ERROR.

dataFileName	IsOK
dissolved_oxygen_measures.csv	FALSE
smell.csv	FALSE
abundances.csv	FALSE

2.3 Warnings

```
print(result, level = 2, listLevel = 20, type = "details", error = 2)
```

2.4 Notes

```
print(result, level = 2, listLevel = 20, type = "details", error = 1)
```

2.4.0.1 values in suggestedValues - note

The details are a table of the same dimension as the input (green) area of the meatadata sheet. The following values are possible:

```
FALSE: If the cell value is not contained in the suggestedValues list.
TRUE : If the cell value is contained in the suggestedValues list.
NA   : empty cell or no suggested values specified
```

One or more FALSE values will result in a WARNING.

speciesID	colour	density	functionalGroup	comment
FALSE	FALSE	FALSE	TRUE	FALSE
FALSE	FALSE	TRUE	FALSE	TRUE

2.4.0.2 values in suggestedValues - note

The details are a table of the same dimension as the input (green) area of the meatadata sheet. The following values are possible:

```
FALSE: If the cell value is not contained in the suggestedValues list.
TRUE : If the cell value is contained in the suggestedValues list.
NA   : empty cell or no suggested values specified
```

One or more FALSE values will result in a WARNING.

treatmentID	treatmentLevelHeight	comment
FALSE	FALSE	TRUE
FALSE	FALSE	TRUE
FALSE	FALSE	TRUE
FALSE	FALSE	TRUE
FALSE	FALSE	TRUE

2.4.0.3 values in suggestedValues - note

The details are a table of the same dimension as the input (green) area of the meatadata sheet. The following values are possible:

```
FALSE: If the cell value is not contained in the suggestedValues list.
TRUE : If the cell value is contained in the suggestedValues list.
NA   : empty cell or no suggested values specified
```

One or more FALSE values will result in a WARNING.

measurementID	variable	method	unit	object	noOfSamplesInTimeSeries	samplingVolume	dataExtractionID	measuredFrom	comment
FALSE	FALSE	TRUE	TRUE	TRUE	FALSE	FALSE	FALSE	FALSE	TRUE
FALSE	TRUE	FALSE	TRUE	TRUE	FALSE	FALSE	FALSE	FALSE	TRUE
FALSE	FALSE	FALSE	FALSE	TRUE	FALSE	FALSE	FALSE	FALSE	TRUE
FALSE	TRUE	FALSE	FALSE	FALSE	FALSE	FALSE	FALSE	FALSE	TRUE

2.4.0.4 values in suggestedValues - note

The details are a table of the same dimension as the input (green) area of the meatadata sheet. The following values are possible:

```
FALSE: If the cell value is not contained in the suggestedValues list.
TRUE : If the cell value is contained in the suggestedValues list.
NA   : empty cell or no suggested values specified
```

One or more FALSE values will result in a WARNING.

dataExtractionID	method	parameter	value	comment
FALSE	TRUE	TRUE	TRUE	FALSE

2.5 OK

```
print(result, level = 2, listLevel = 20, type = "details", error = 0)
```

2.5.1 Structural / Formal validity - OK

x
TRUE

2.5.2 Experiment - OK

The details are a table with one row per unique validation. The column `Module` contains the name of the validation, The column `error` contains the actual error of the validation. The following values are possible for the column `isTRUE`:

```
TRUE : If the validation was `OK`.
FALSE: If the validation was an `error`, `warning` or `note`.
NA   : If at least one v alidation resulted in `NA
```

One or more FALSE or missing values values will result in an ERROR.

Module	errorCode	isOK
types	OK	TRUE

2.5.2.1 conversion of values into specified type lossless possible - OK

The details are a table of the same dimension as the input (green) area of the meatadata sheet. The following values are possible:

```
FALSE: If the cell contains an error, i.e. can not be losslessly converted.
TRUE : If the cell can be losslessly converted and is OK.
NA   : empty cell
```

One or more FALSE values will result in an ERROR.

name	temperature	light	humidity	incubator	container	microcosmVolume	mediaType	mediaConcentration	cultureConditions	comunityType	mediaAdditions	duration	cor
TRUE	TRUE	TRUE	TRUE	TRUE	TRUE	TRUE	TRUE	TRUE	TRUE	TRUE	TRUE	TRUE	NA

2.5.2.2 conversion of values into specified type lossless possible - OK

The details are a table of the same dimension as the input (green) area of the meatadata sheet. The following values are possible:

```
FALSE: If the cell contains an error, i.e. can not be losslessly converted.
TRUE : If the cell can be losslessly converted and is OK.
NA   : empty cell
```

One or more FALSE values will result in an ERROR.

speciesID	colour	density	functionalGroup	comment
TRUE	TRUE	TRUE	TRUE	TRUE
TRUE	TRUE	NA	TRUE	NA

2.5.2.3 ID Field present and in the first column - OK

Returns a boolean value, with the following possible values:

```
TRUE  : The tab's first column is an ID field
FALSE : The tab's first column is not an ID field
```

FALSE will result in an ERROR.

hasIDField	isOK
tab has ID field in first column	TRUE

2.5.2.4 conversion of values into specified type lossless possible - OK

The details are a table of the same dimension as the input (green) area of the meatadata sheet. The following values are possible:

```
FALSE: If the cell contains an error, i.e. can not be losslessly converted.
TRUE : If the cell can be losslessly converted and is OK.
NA   : empty cell
```

One or more FALSE values will result in an ERROR.

treatmentID	treatmentLevelHeight	comment
TRUE	TRUE	NA
TRUE	TRUE	NA
TRUE	TRUE	NA
TRUE	TRUE	NA
TRUE	TRUE	NA

2.5.2.5 ID Field present and in the first column - OK

Returns a boolean value, with the following possible values:

```
TRUE  : The tab's first column is an ID field
FALSE : The tab's first column is not an ID field
```

FALSE will result in an ERROR.

hasIDField	isOK
tab has ID field in first column	TRUE

2.5.2.6 conversion of values into specified type lossless possible - OK

The details are a table of the same dimension as the input (green) area of the meatadata sheet. The following values are possible:

```
FALSE: If the cell contains an error, i.e. can not be losslessly converted.
TRUE : If the cell can be losslessly converted and is OK.
NA   : empty cell
```

One or more FALSE values will result in an ERROR.

measurementID	variable	method	unit	object	noOfSamplesInTimeSeries	samplingVolume	dataExtractionID	measuredFrom	comment
TRUE	TRUE	TRUE	TRUE	TRUE	TRUE	TRUE	TRUE	TRUE	NA
TRUE	TRUE	TRUE	TRUE	TRUE	TRUE	TRUE	TRUE	TRUE	NA
TRUE	TRUE	TRUE	TRUE	TRUE	TRUE	TRUE	TRUE	TRUE	NA
TRUE	TRUE	TRUE	TRUE	TRUE	TRUE	TRUE	TRUE	TRUE	NA

2.5.2.7 ID Field present and in the first column - OK

Returns a boolean value, with the following possible values:

```
TRUE  : The tab's first column is an ID field
FALSE : The tab's first column is not an ID field
```

FALSE will result in an ERROR.

hasIDField	isOK

hasIDField	isOK
tab has ID field in first column	TRUE

2.5.2.8 conversion of values into specified type lossless possible - OK

The details are a table of the same dimension as the input (green) area of the meatadata sheet. The following values are possible:

```
FALSE: If the cell contains an error, i.e. can not be losslessly converted.
TRUE : If the cell can be losslessly converted and is OK.
NA   : empty cell
```

One or more FALSE values will result in an ERROR.

dataExtractionID	method	parameter	value	comment
TRUE	NA	NA	NA	TRUE

2.5.2.9 ID Field present and in the first column - OK

Returns a boolean value, with the following possible values:

```
TRUE  : The tab's first column is an ID field
FALSE : The tab's first column is not an ID field
```

FALSE will result in an ERROR.

hasIDField	isOK
tab has ID field in first column	TRUE

2.5.2.10 conversion of values into specified type lossless possible - OK

The details are a table of the same dimension as the input (green) area of the meatadata sheet. The following values are possible:

```
FALSE: If the cell contains an error, i.e. can not be losslessly converted.
TRUE : If the cell can be losslessly converted and is OK.
NA   : empty cell
```

One or more FALSE values will result in an ERROR.

dataFileName	columnName	columnData	mappingColumn	type	description	comment
TRUE	TRUE	TRUE	NA	TRUE	NA	NA
TRUE	TRUE	TRUE	TRUE	TRUE	NA	NA
TRUE	TRUE	TRUE	NA	TRUE	NA	NA
TRUE	TRUE	TRUE	NA	TRUE	NA	NA
TRUE	TRUE	TRUE	NA	TRUE	NA	NA
TRUE	TRUE	TRUE	NA	TRUE	TRUE	NA
TRUE	TRUE	TRUE	TRUE	TRUE	NA	NA
TRUE	TRUE	TRUE	NA	TRUE	NA	NA
TRUE	TRUE	TRUE	NA	TRUE	NA	NA
TRUE	NA	TRUE	TRUE	TRUE	NA	NA
TRUE	TRUE	TRUE	TRUE	TRUE	NA	NA
TRUE	TRUE	TRUE	NA	TRUE	TRUE	NA
TRUE	TRUE	TRUE	TRUE	TRUE	NA	NA
TRUE	TRUE	TRUE	NA	TRUE	NA	NA
TRUE	NA	TRUE	TRUE	TRUE	NA	NA
TRUE	TRUE	TRUE	NA	TRUE	NA	NA
TRUE	TRUE	TRUE	NA	TRUE	TRUE	NA
TRUE	TRUE	TRUE	TRUE	TRUE	NA	NA
TRUE	TRUE	TRUE	NA	TRUE	NA	NA
TRUE	TRUE	TRUE	TRUE	TRUE	NA	NA

3 Structure info

```
attributes(x)
```

```
## $names
##  [1] "error"               "details"            "header"
##  [4] "description"         "descriptionDetails" "comment"
##  [7] "structure"           "Experiment"         "Genus"
## [10] "Treatments"          "Measurement"        "DataExtraction"
## [13] "DataFileMetaData"
##
## $class
## [1] "dmdScheme_validation" "list"
```

4 TODO

- Use conditional text to explicitly say pass or fail, so that users don't have to read the R output (or at least get lovely green text for pass, and red for fail!)
- Validation out of attempt to parse date variables
